# On the Shape-Selected, Ligand-Free Preparation of Hybrid Perovskite (CH_3_NH_3_PbBr_3_) Microcrystals and Their Suitability as Model-System for Single-Crystal Studies of Optoelectronic Properties

**DOI:** 10.3390/nano11113057

**Published:** 2021-11-13

**Authors:** Ulrich Johannes Bahnmüller, Henning Kuper, Tobias Seewald, Yenal Yalҫinkaya, Jörg August Becker, Lukas Schmidt-Mende, Stefan A. L. Weber, Sebastian Polarz

**Affiliations:** 1 Institute of Inorganic Chemistry, Leibniz University Hannover, Callinstraße 9, 30167 Hannover, Germany; ulrich.bahnmueller@aca.uni-hannover.de; 2 Institute of Physical Chemistry and Electrochemistry, Leibniz University Hannover, Callinstraße 3A, 30167 Hannover, Germany; henning.kuper@pci.uni-hannover.de (H.K.); Joerg-august.becker@pci.uni-hannover.de (J.A.B.); 3 Department of Physics, University of Konstanz, Universitätsstraße 10, 78457 Konstanz, Germany; tobias.seewald@uni-konstanz.de (T.S.); Lukas.schmidt-mende@uni-konstanz.de (L.S.-M.); 4 Max-Planck-Institute for Polymeric Research, Ackermannweg 10, 55128 Mainz, Germany; yalcinkayay@mpip-mainz.mpg.de (Y.Y.); webers@mpip-mainz.mpg.de (S.A.L.W.); 5 Institute of Physics, Johannes Gutenberg University Mainz, Staudingerweg 7, 55128 Mainz, Germany

**Keywords:** hybrid perovskites (HYPE), methylammonium lead bromide, aerosol synthesis, shape-related properties, optoelectronic properties of MAPbBr_3_

## Abstract

Hybrid perovskite materials are one of the most promising candidates for optoelectronic applications, e.g., solar cells and LEDs, which can be produced at low cost compared to established materials. Although this field of research has seen a huge upsurge in the past decade, there is a major lack in understanding the underlying processes, such as shape-property relationships and the role of defects. Our aerosol-assisted synthesis pathway offers the possibility to obtain methylammonium lead bromide (MAPbBr_3_) microcrystals from a liquid single source precursor. The differently shaped particles are aligned on several substrates, without using a directing agent or other additives. The obtained particles show good stability under dry conditions. This allows us to characterize these materials and their pure surfaces at the single-crystal level using time- and spatially resolved methods, without any influences of size-dependent effects. By optimizing the precursor for the aerosol process, we were able to eliminate any purification steps and use the materials as processed. In addition, we performed theoretical simulations to deepen the understanding of the underlying processes in the formation of the different crystal facets and their specific properties. The model system presented provides insights into the shape-related properties of MAPbBr_3_ single crystals and their directed but ligand-free synthesis.

## 1. Introduction

Nanomaterials and nanocomposites have seemingly superseded “traditional” compounds in almost all different fields and applications. Their properties are undoubtedly fascinating, but the number of nanomaterials that have made it to the market already, is still small. Using the example of nanomaterials in photovoltaics, we can discuss possible reasons for some of the associated problems. Despite a large number of candidates and decades of research into new materials, silicon is still by far the dominant compound in the solar cell industry [1,2,3]. One of the alternatives for silicon is the class of the so-called hybrid perovskites e.g., CH_3_NH_3_PbHal_3_ (Hal = I, Br) [4,5,6,7]. Metal-halide perovskite semiconductors show extraordinary optoelectronic properties, such as high luminescence yields, sharp absorption onsets, and long carrier lifetimes. Since their discovery as a promising semiconductor material a few years ago [8,9,10], the efficiency of perovskite solar cells has now reached over 25% [11]. Although the number of material compositions and morphologies for high-performance cells has increased dramatically over the years, there are still general questions that remain unresolved. A major problem is that studies on solution-processed perovskite solar cells show a large variation in solar cell parameters (short-circuit current density (JSC), fill factor (FF) and open circuit voltage (VOC)), and also their photoluminescence and charge diffusion properties.

One possible explanation for the uncertainty in hybrid perovskites in particular, and also many in emerging optoelectronic materials in general, is that the properties are dominated by defects, sometimes more, sometimes less. Since defects are caused by entropy and are thus hardly controllable, difficult to detect and quantify, properties and also material quality are scattered. There is evidence that important optoelectronic properties are affected by grain boundaries, crystal defects, and crystal orientation. Defects exist, for example as interstitials, lattice defects, and vacancies and are involved in non-radiative loss mechanisms that limit device performance. In addition, the stability and hysteresis effect of perovskite solar cells are believed to depend to some extent on the structural parameters, often in combination with effects at interface layers. Theoreticians have calculated the possible termination of the perovskite film, the probability of formation of defects and their influence on the performance of perovskite solar cells [12]. Haruyama concluded from the calculations that flat terminations, saturated by the addition of excess PbI_2_, are less stable than vacant terminations on all surface directions [13]. Wang et al. demonstrated with first-principle DFT calculations for stoichiometric surfaces that their stability depends mainly on the coordination number of the surface atoms [14]. The theoretical results encourage experimental verification to understand the role of surface terminations and thus the importance of the surface direction [15].

Experimental studies dealing with the aforementioned effects have been carried out on fully assembled and very defective and complex systems [16]. Extensive research has investigated the properties and nature of such defects in perovskites using films and nanostructures in solution, focusing on measurements of defect ensembles at macroscopic length scales [17,18,19]. These studies have so far accepted the defects as intrinsic properties of the material, with efforts mostly focusing on the eliminating of the defects and “passivating” the impact of defects on carrier dynamics [20,21,22]. Ono and Qi summarized experimental results on the surface and interface aspects in their review [23]. Studies on polycrystalline films make it necessary to investigate the local properties and morphologies [24]. With Kelvin probe force microscopy (KPFM) measurements [25], the electrical surface potential distribution between crystal grains/grain boundaries [26] and across different layers of a solar cell [27,28,29,30] can be determined with a lateral resolution of 10–50 nm. In addition, local conductivity, current-voltage [31,32], and photoluminescence measurements [20,33,34,35] are possible, providing information on the role of grain boundaries. Significant differences in photoluminescence efficiency were observed between the different grains, suggesting that their orientation and specific structure has a strong influence on device performance. The defect densities vary in different grains, which might originate from the different grain direction and their different surface properties. It has also been observed that charge diffusion can vary across grain boundaries, which affects the overall electronic transport properties of the perovskite film [34]. It has been reported that there are band bends at the grain boundaries that appear to attract predominantly photo-induced electrons and could act as an interface for charge dissociation [32]. Polycrystalline layers, as used in solar cells, thus exhibit a great heterogeneity in terms of electronic and photophysical properties. It can be concluded that it is very difficult to determine the specific role of defects on optoelectronic properties and solar cell parameters.

The needed, fundamental studies on defect-property correlations in metal-halide perovskites would benefit from much more defined model systems. To overcome the challenges connected to the polycrystallinity of the films, researchers have started to investigate single crystal perovskites [15]. Having demonstrated crystal formation by reverse temperature crystallization, the Bakr group used this synthetic approach to obtain macroscopic single crystals and study their optical properties. They found that an increase in temperature leads to crystallization of perovskite materials from liquid precursor solutions, using coordinating solvents such as DMF [36]. They investigated the stability of these materials and the behavior of charge carriers and compared them with the more widely used polycrystalline films. In their studies, they found that single crystals have better optical properties due to their lower defect density and the associated longer charge carrier diffusion [37]. In another publication, the contrast between the surface and bulk states of hybrid perovskite single crystals was illustrated [38]. They also summarized the great benefits of single crystalline hybrid perovskite materials for their application as semiconductors in light conversion applications [39]. Another extensive work comparing the recombination kinetics of bulk and surface was carried out by Wu et al. on macroscopic single crystalline MAPbBrX_3_ materials [40]. Due to the growing interest in nonlinear optical applications of hybrid perovskite materials, Kriso et al. worked on single crystalline materials to gain insights into the nonlinear refraction by focusing on the nonlinear refractive index of these materials [41]. In a joint experimental and theoretical approach, She et al. investigated MA–iodine-terminated (001) surfaces of the orthorhombic perovskite films grown on Au(111) surfaces by scanning tunnelling microscopy [15]. They found surface iodine dimerization due to the realignment of the surface MA dipoles, which enhance the interactions with the surface iodine anions. Gao et al. used single crystal nanowires as photodetectors [42]. They could improve the sensitivity significantly by introducing oleic acid soaking to passivate surface defects of MAPbI_3_ nanowires. This example shows that the termination of the perovskite has strong influence on the device characteristics. deQuilettes et al. [34] used correlated confocal and wide-field fluorescence microscopy to investigate the role of grains on charge carrier recombination and transport. The charge carriers are subject to anisotropic diffusion due to the different connections between the individual grains. Local variations in non-radiative recombination are the main reason for the observed photoluminescence heterogeneity in the films. These few experimental examples clearly show the importance of a systematic study of the physical properties of high definition perovskite crystals to understand the role of crystallinity, crystal orientation, defects, and the presence of impurities and dopants on the optoelectronic properties.

**Figure 1:**
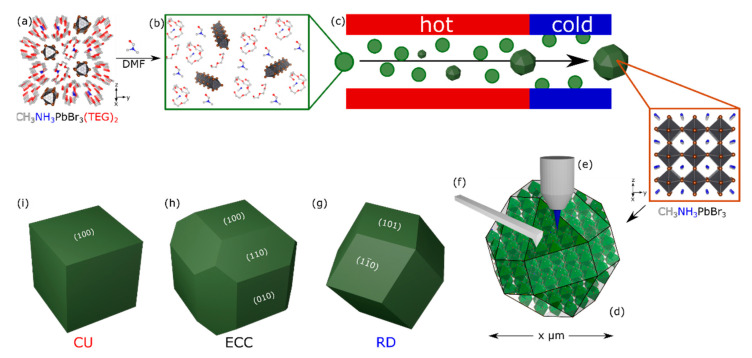
(**a**) Structure of the hybrid perovskite single-source precursor [43]. (**b**) Structural break-up of [PbBr_3_]^−^_∞_ chains by DMF addition. (**c**) Injection of a spray of the precursor into the aerosol setup and transformation into micrometer sized particles of shape-controlled CH_3_NH_3_PbBr_3_ single-crystals by inverse temperature crystallization. (**d**) Individual particles are investigated by spatially resolved methods such as μ-photoluminescence (PL). (**e**) μ-transient absorption spectroscopy (TAS) and (**f**) Kelvin-probe force microscopy (KPFM). The three hybrid perovskite microcrystal morphologies covered in this paper and the set of symmetry-equivalent surfaces in Miller-index notation: (**g**) rhombic dodecahedron (RD), (**h**) edge cut cube (ECC), and (**i**) cube (CU).

The single crystals described in the last paragraph have macroscopic dimensions compared to the nano- to micrometer-sized domains found in hybrid perovskite thin films, which are technically most relevant for photovoltaics. To fill this gap, we propose that single-crystal microcrystals are an ideal model system when it is possible to perform physical studies at the single-particle level (see Figure 1). They offer the possibility to characterize single surface facets, not being influenced by size-dependent effects, as the quantum size effect for nanoparticles, or bulk properties for macroscopic single crystals.

The task for our current work is therefore the production of microcrystals with single crystal character and their subsequent physical characterization with ensemble methods in comparison to spatially resolved techniques. There are several aspects to consider for such a system. Previous literature has argued that the shape of a crystal, whether it is a nano-, micro-, or macrocrystal, correlates with a certain set of surfaces that corresponds to certain lattice planes (hkl) [44]. One way of approaching the photophysical properties of certain facets is therefore to produce differently shaped microcrystals, as shown in Figure 1g–i. The success in probing single microcrystals, in particular using KPFM, requires that the particles are positioned in an isolated, non-agglomerated form on a suitable substrate. Furthermore, their surfaces have to be clean, meaning consisting of the pure perovskite. Standard recipes for the preparation of small crystals involve the use of organic ligands, which stabilize the surfaces energetically [45,46,47]. However, these ligands remain attached to the crystal forming an insulating layer, which may also have an electronic effect on the interface region [48,49]. It can be concluded that an additional requirement is the synthesis method that yields perovskite microcrystals with clean surfaces. This offers the possibility to fine-tune the optoelectronic properties through the synthetic parameters alone, resulting in different morphologies without changing the composition of the material [50]. 

## 2. Materials and Methods

Extra dry *N*,*N*-dimethylformamide (DMF) (99.8%) was purchased from fisher scientific. Triethyleneglycole (TEG), HBr and methylammonium (MA) solutions, and PbBr_2_ (≥98%) were purchased from Sigma Aldrich (St. Louis, MO, USA). MABr was synthesized from solutions of HBr and MA according to a literature-known procedure [51].

All syntheses were carried out under inert gas atmosphere. TEG and all solid chemicals were dried under vacuum at 65 °C for at least 24 h before being used.

The liquid precursors were prepared according to literature, by solving MABr and PbBr_2_ in the appropriate solvent [36,43]. The precursor solutions used for the aerosol synthesis were mixed in a 1:1 (*v*:*v*) ratio of both solvents and 0.3 M for PbBr_2_.

The detailed procedure is explained exemplarily for the equimolar MA:Pb-eq precursor solutions in TEG or DMF, for the excess MABr precursor solutions the same procedure was applied, using larger amounts of MABr (1.3 eq and 1.8 eq). In a typical synthesis 550.5 mg PbBr_2_ were dissolved in 5 mL TEG. The solution was stirred for 72 h at 65 °C under high vacuum (hv). Afterwards the solution was cooled to room temperature (rt) under N_2_ and further cooled down to 5 °C for at least 1 h. Total of 168 mg MABr was added to the cooled solution and dissolved gradually using a vortex mixer RS-VA 10 from phoenix instruments. The obtained clear and colorless solution was stored at rt. For the DMF-based precursor solution 168 mg MABr were dissolved in 5 mL DMF. Afterwards 550.5 mg PbBr_2_ were added and dissolved using a vortex mixer. The obtained clear, colorless solution was stored at rt. For the aerosol process the TEG-based precursor solution is mixed with a DMF-based precursor solution 1:1 (*v*/*v*).

The material synthesis via an aerosol process is carried out in a tubular oven using constant nitrogen flow (2 L/min) as carrier gas. The aerosol is generated using a reservoir vessel equipped with a suction tube, connected to the aerosol generator (model 3076, TSI Inc., Shoreview, MN, USA). For the generation of perovskite particles, a temperature of 150 °C was applied. The crystals were collected on different substrates, (FTO- or glass-slides; silicon wafer) depending on the characterization methods applied, and were used without further treatment. Prior to particle deposition, the substrates were treated with oxygen plasma for 10 min using a Femto plasma cleaner (Diener electronic GmbH & Co. KG, Ebhausen, Germany). For a typical aerosol synthesis, 5 mL of precursor was used, resulting in a reaction time of 90 min.

SEM images were obtained from particles collected on silicon substrates, without further processing, using a JSM-6700F microscope (JEOL Ltd., Akishima, Japan). For UV/Vis measurements the particles were collected on a glass substrate and used as processed. They were measured using an Cary 5000 spectrometer (Agilent Technologies Inc., Santa Clara, CA, USA) with an integrating sphere. For evaluation of the bandgap, the Tauc plot was plotted from the absorption data. Time-resolved photoluminescence (tr-PL) measurements were obtained using a FluoTime 300 spectrometer (PicoQuant GmbH, Berlin, Germany). Space-resolved TAS (μTAS) and PL (μPL) were measured of particles collected on glass substrates without further processing. The set-up is built up by a RegA900 amplifier seeded by a Mira900 (both from Coherent Inc., Santa Clara, CA, USA), both by coherent, 800 nm, 200 fs. For 1-photonabsorption (1PA) and TAS measurements the second harmonic was used for excitation, for 2-photonabsorption (2PA) the fundamental was used. For TAS probe a portion of the fundamental was focused into a sapphire plate to generate a white light continuum, starting at 450 nm and cut off at 700 nm by a short pass filter. For spatial resolution light was collected by a 100× objective and detected with an sCMOS camera Zyla5.5 attached to an imaging spectrograph Kymera193i(both by Andor Technology Ltd., Belfast, UK).

The UV/Vis kinetics were measured using a Cary 4000 with Praying Mantis diffuse reflectance accessory (Agilent Technologies Inc.). To control the temperature of the sample a heater with a WATLOW series 999 control unit (Harrick Scientific Products Inc., Pleasantville, NY, USA) was used.

PXRD measurements were performed using a Theta/Theta diffractometer (STOE & Cie GmbH, Darmstadt, Germany) with Θ-Θ geometry using Cu Kα (1.540598 Å) from a PW2273/20 X-ray source (Malvern Panalytical, Eindhoven, Netherlands). Carried out in relfexion, a graphite secondary monochromator was used before the scintillation counter, which was used to detect the signal.

KPFM measurements for all samples were carried out on an MFP 3D AFM (Asylum Research/Oxford Instruments, Santa Barbara, CA, USA) in a nitrogen-filled glovebox with the humidity level around 0.9%. Pt/Ir-coated SCM-PIT-V2 cantilevers (Bruker Corporation, Billerica, MA, USA) with 70 kHz resonance frequency and 2 N7m spring constant were employed as scanning probes. In order to perform KPFM feedback, HF2 Lock-In amplifier (Zurich Instruments Corporation, Zurich, Switzerland) and the KPFM drive voltage were applied to the tip. The work functions were calculated by the following equation:

*U*_CPD_ = (Φ_tip_ − *Φ*_sample_)/*e* (1)

where *U*_CPD_, Φ_tip_, *Φ*_sample_, and *e* are measured CPD signal, work function of the tip, work function of the sample, and the elemental charge, respectively.

For the quantitative KPFM measurements for exact work functions, a HOPG reference with the work function of 4.475 ± 0.005 eV was used before and after perovskite crystal measurements [52]. The HOPG was freshly cleaved inside the nitrogen-filled glovebox prior to the experiment.

## 3. Results and Discussion

### 3.1. Aerosol Synthesis of Shape-Controlled Perovskite Microcrystals

Recently, we have gained substantial experience in the past with a novel single-source precursor system for hybrid perovskites (Figure 1b) [43,48,49,53,54,55]. The precursor is obtained by reacting PbX_2_ (X = Br, I) with an equimolar amount of CH_3_NH_3_X and ethylene glycol derivatives such as TEG (tri-ethylene glycol; C_6_H_14_O_2_). The precursor contains [PbBr_3_]^−^_∞_ chains with CH_3_NH_3_^+^ as a counter-cation wrapped by two TEG groups (Figure 1a). It is solid at rt and melts at *m_p_* = 35 °C. This melt was used in ref. [43] for the preparation of MAPbBr_3_ microcrystals via an aerosol-assisted synthesis pathway. We started to reproduce the latter results, but we saw that the quality of the samples was not sufficient for the planned photophysical studies. The following problems were encountered. The fraction of particles was small, which were separated on the substrate. The majority of particles was strongly aggregated (Supporting information Figure S1a). Many microcrystals are surrounded by smaller structures (Figure S1b). The same structures can be obtained by depositing a liquid precursor, if the formation of microcrystals in the aerosol is prevented by lowering the temperature in the decomposition zone of the precursor (Figure S1c). No reflections were detected by powder X-ray diffraction (PXRD) analysis, indicating that the compound is either amorphous or has not been converted to a solid material at all (Figure S1d). Crystallization occurred post-treatment and resulted in MAPbBr_3_. Energy dispersive X-ray spectroscopy (EDX) confirms the composition of lead and bromide (Figure S1e). Since carbon, oxygen, and nitrogen are known to be barely visible in EDX spectra, implicating that the liquid precursor is merely condensed on the substrates. This confirms the previously mentioned assumption that crystallization is caused by post-treatment, e.g., the high vacuum in the SEM evaporating the liquid components of the precursor. Therefore, it can be concluded that the impurities surrounding the microcrystals in Figure S1b are caused by unreacted precursor melt, which solidifies on the substrate forming MAPbBr_3_. In general, it is unfavorable if only a part of the precursor is converted into perovskite microcrystals during synthesis, since any impurity may have an undesirable effect on the photophysical measurements.

**Figure 2:**
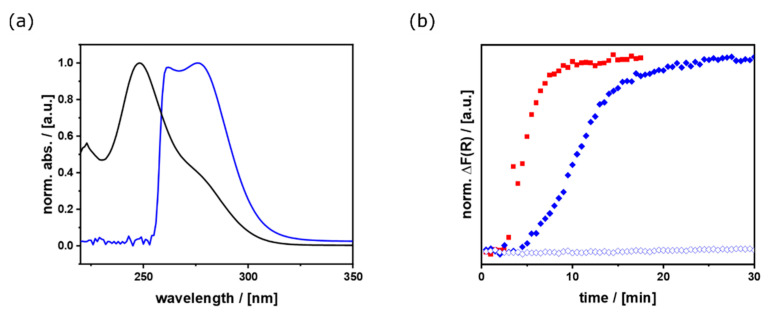
(**a**) UV/Vis absorbance spectra for the equimolar (MA:Pb) precursor systems with pure TEG (black) and 1:1 (*v*:*v*) mixture of TEG and DMF (blue); (**b**) UV/Vis kinetics for the three different precursor systems from time-dependent measurements while heating the precursors from 35 up to 75 °C (heat rate 16 °C/min), red (1.8 eq MABr in TEG-DMF; aborted after ~17 min, because no further changes were observed), solid blue (1 eq MABr in TEG-DMF), and clear blue (1 eq MABr in TEG).

The following adjustments were made to solve the problems. It can be seen from Figure 1a–c that the liquefaction and transformation of the precursor into perovskite crystals require the breaking of the [PbBr_3_]^−^_∞_ chains and the release of CH_3_NH_3_^+^ from coordination with TEG. Therefore, we tried to increase the reactivity of the precursor by the addition of dimethylformamide (DMF). The effect of the presence of DMF was investigated by UV/Vis spectroscopy (Figure 2a). In comparison to pure [CH_3_NH_3_TEG_2_]PbBr_3_ (*λ*_max_ = 250 nm) as a reference, there is a characteristic red-shift and change in the absorption bands. In agreement with the literature [56,57], the higher intensity for the signal at 276 nm in the DMF containing system can be explained by a shortening of the [PbBr_3_]^−^_∞_ chains. The signal is related to the PbBr_4_^2−^ species, which terminate the chains (Figure S2). The superposition of two absorption bands (*λ*_max_ = 262, 276 nm) for the DMF containing precursor can be explained by the different PbBr*_x_*^(*x*−2)−^ species in solution (Figure 2a) [57]. The kinetic measurements (Figure 2b) show the comparison of precursor solutions with either different solvents (solid vs. clear blue curve) or different concentrations of salts (red vs. blue). The increase in Δ*F*(*R*) is indicative for the conversion of the liquid precursor into the perovskite material, showing a band edge in the UV/Vis spectra (Figure S3a–c). The kinetics confirm the faster reaction depending on the MABr concentration, which is in agreement with previous reports [43,57,58]. Furthermore, the measurements confirm that the addition of DMF accelerates the reaction time, which can also be explained by the previously mentioned shortening of the [PbBr_3_]^−^_∞_ chains, leading to more reactive terminal PbBr_4_^2−^ groups. While no turnover was observed with the pure TEG system (Figure 2b), light blue) under the applied conditions, the system mixed with DMF shows a clear turnover after about 10 min using the same salt concentrations (equimolar MABr to PbBr_2_ ratio). From this, it can be concluded that, in addition to the already known influence of Br^−^, the combination of the two coordinating solvents TEG and DMF also leads to a shortening of the [PbBr_3_]^−^_∞_ chains and therefore accelerates the crystal formation.

**Figure 3:**
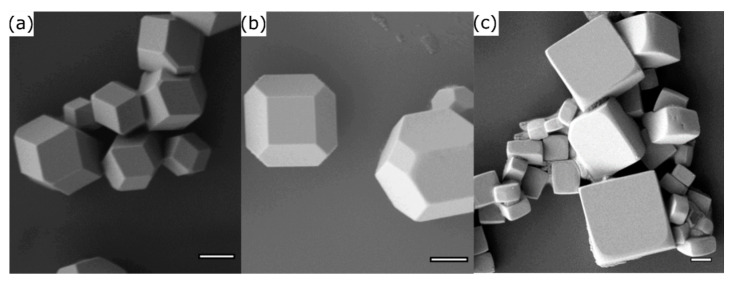
SEM images of the (**a**) RD microcrystals, (**b**) ECC microcrystals formed using an 30% excess of CH_3_NH_3_Br, and (**c**) CU microcrystals using an 80% excess of CH_3_NH_3_Br; scale bars = 1 μm.

In addition, we tried to remove possibly the unreacted aerosol droplets of the precursor by condensation on the cold glass wall in the cold zone at the end of the reactor (Figure 1c). Examination of the resulting sample shows that exclusively microcrystals were deposited on the substrates (Figure 3a) and precursor residues are absent. The morphology of the microcrystals resembles a rhombic dodecahedron (RD; see also Figure 1g). The microcrystals are terminated by the (101), ($1\bar{1}0)$ lattice planes and its symmetry equivalents. It is important to note that there are no (100) faces in this morphology. The RD microcrystals resulted from DMF∙[CH_3_NH_3_TEG_2_]PbBr_3_, respectively from the precursor system with equimolar amount of MABr compared to PbBr_2_ to form the perovskite material. If an excess of MABr in relation to PbBr_2_ is used, the morphology of the microcrystals can be varied. For an excess of 30% the formed particles resemble edge-cut cubes (ECC; Figure 3b). An even higher excess of MABr (80%) results in the formation of cubic particles (CU; Figure 3c). The latter two morphologies contain (100) surfaces (Figure 1h,i).

The order of formation of these particles is surprising because the (100) surfaces are the most stable ones and therefore a morphology like RD without such surfaces cannot be the thermodynamically preferred, the Wulff shape, of the MAPbBr_3_ material [59]. The Wulff form (CU) should have formed spontaneously, and other shapes may have been formed if one changes the formulation, e.g., by adding potential ligands, which was shown for the inorganic perovskite SrTiO_3_ [60]. There are two possibilities to explain the result. A first idea is that MA^+^ in the excess MABr preferentially binds to the (100) surfaces, further stabilizing them. However, (100) is already the most stable surface and there is no evidence in literature that MA^+^ can be selective. Therefore, it is much more likely that RD (and ECC) are morphologies produced by kinetically controlled reaction pathways. If this hypothesis is correct, the addition of MABr would also influence the kinetics of crystallization of the hybrid perovskite. As mentioned above, Figure S3d shows the influence of an excess of MABr on the UV/Vis spectrum of the DMF precursor. The absorption band (*λ*_max_ = 276 nm) is characteristic for a higher fraction of terminal PbBr_4_^2−^ units [57] and, thus, shorter chains become even more pronounced. This leads to the assumption that terminal PbBr_4_^2−^ units are more reactive than bridging PbBr_3_^−^ units, the presence of excess MABr is expected to accelerate the formation of the hybrid perovskite. Furthermore, this is totally in accordance with the previously discussed kinetic measurements for the different precursor compositions (Figure S3a–c). In other words, the kinetic barriers are lowered when additional MABr is present, which favors the formation of the Wulff form. This hypothesis can also be confirmed by thermodynamic considerations in connection with the incorporated theoretical ab-initio calculations. The underlying theory and the results of these simulations will be discussed in detail later. Here it is important to mention that these considerations suggest which morphology forms depend on the activity of the lead bromide species of the precursor. The increasing MABr content in the precursor leads to an increase in activity, which explains the shift toward the preferential formation of (100) surfaces representing the Wulff shape. This is fully consistent with the experimental observations conducted in this study and discussed previously.

**Figure 4:**
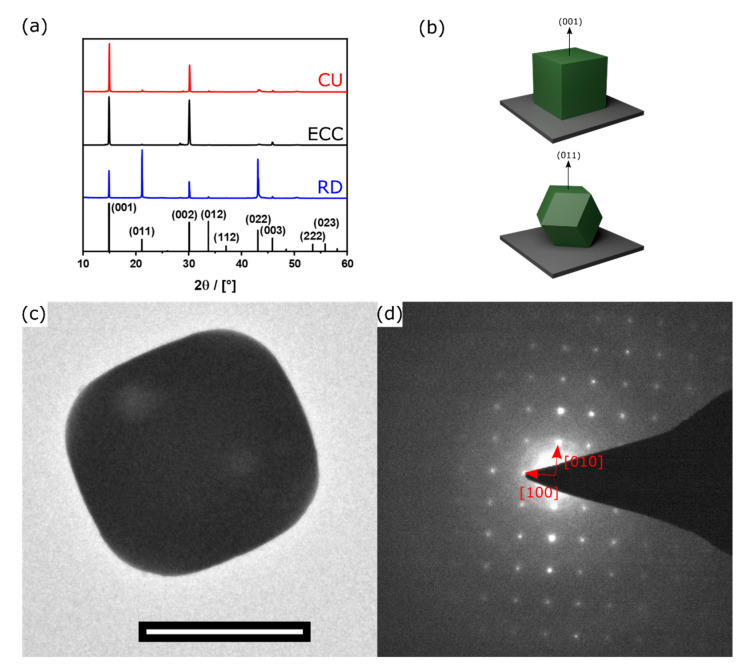
(**a**) PXRD pattern from RD (blue), ECC (black), and CU (red) microcrystal and the reference pattern of CH_3_NH_3_PbBr_3_ (black; No. 252415.cif) [62]; (**b**) Orientation of CU- (top) and RD- (bottom) microcrystals on the substrate; (**c**) TEM micrograph (scale bar = 500 nm); and (**d**) ED of one single CU-microcrystal.

The size of the microcrystals was determined by a statistical evaluation of SEM-data. As expected for an aerosol method, there is a polydisperse distribution of sizes, ranging from several 100 nm to several μm (Figure S4a). However, it can be seen that the presence of an excess MABr has almost no effect on the average size of the microcrystals (*D_av_*). This means that the physical data will also be comparable to each other. In particular, the surface-to-volume ratio of RD- (*D_av_* = 1.4 ± 0.5 μm) is very comparable to that of CU microcrystals (*D_av_* = 1.8 ± 0.6 μm) (Figure S4b). A detailed characterization is shown exemplarily for the CU-perovskite microcrystals. Figure 4a shows a powder X-ray diffraction pattern of the sample measured in Bragg Brentano geometry. The patterns confirm phase purity and the existence of the MAPbBr_3_ perovskite. However, this particular diffraction geometry is helpful because it can reveal information about the orientation of the particles on the substrate. The dominance of (001) diffraction signal and absence of signals such as (011) in the case of the CU, show that the microcrystal is indicative of an orientation of the particles in this direction parallel to the substrate (Figure 4b). The pattern of the ECC microcrystals shows the same orientation on the substrate, due to the distinct (001)-facets of this morphology. The PXRD pattern of the RD microcrystals are characterized by a significant increase of (011) signals. Since the RD morphology does not contain (001) surfaces, it is obvious that it cannot be deposited on the substrate in the same orientation as the CU particles (Figure 4b). The latter results open a perspective for directional measurements. The orientation of the particles and the single-crystal character can be confirmed by transmission electron microscopy (TEM) and electron diffraction (ED) measurements shown in Figure 4c,d. The ED pattern shows discrete diffraction spots, and the lattice parameter determined from the ED pattern is 5.8326 Å which corresponds to the expected value in the literature [61].

**Figure 5:**
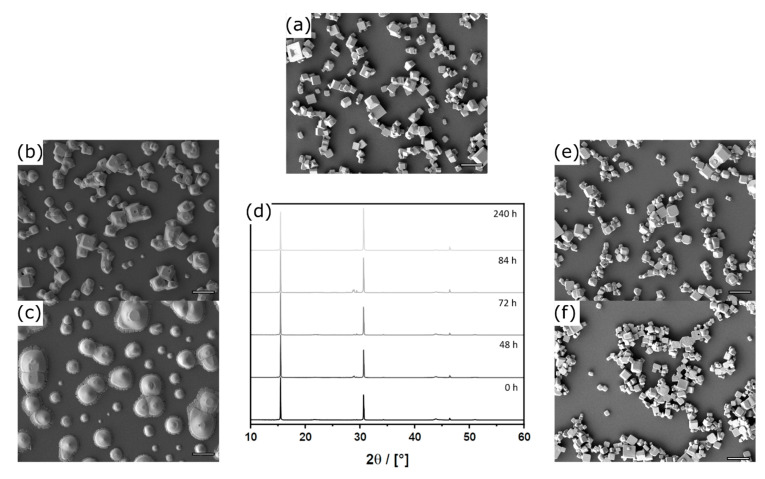
SEM micrographs (scale bars = 5 μm) of the freshly prepared sample with a density of (**a**) CU-microcrystals. Storage under ambient conditions for (**b**) 48 h and (**c**) 240 h and (**d**) corresponding PXRD-patterns with dominant (100) and (200) reflexes. SEM micrographs for storage under dry conditions for (**e**) 48 h and (**f**) 96 h.

Before the optoelectronic properties of the materials can be described and compared, another very important aspect must be mentioned: the stability of the samples. Changes in the morphology will of course lead to artefacts in the physical measurements, and this must be excluded. It is well documented in literature that hybrid perovskite materials are quite sensitive, in particular against humidity [38,63,64,65]. If we store our material (Figure 5a) under ambient conditions, we see that the microcrystals are hardly present after 48 h and have completely disintegrated after 240 h (Figure 5b,c). This morphological deterioration makes selective examination of single facets completely impossible. It is remarkable that there are only insignificant changes in PXRD pattern (Figure 5d). Although the PXRD pattern still confirms the presence of the perovskite phase during the whole degradation process, the relative intensities of the (100) and the (200) reflexes change with increasing storage duration (Figure 5d). This change can be explained by the increasing intercalation of water into the perovskite structure forming a hydrolyzed MA species and therefore increasing the intensity of the (200) reflex. The intercalated H_2_O increases the electron density for the (200) plane, where the MA cation is located, which results in higher intensities in the diffraction pattern. These findings are in common with a work from Christians et al., who studied the influence of humidity on MAPBI_3_ materials in detail [64]. When the particles are stored for 42 d under ambient conditions, the PXRD pattern (Figure S5a) still confirms the presence of the perovskite phase and shows no evidence of crystalline decomposition products, such as PbBr_2_. Although the SEM image (Figure S5c) shows complete decomposition of the particular cubic shape and a darker film like structure on top of the degraded structures, the EDX spectra (Figure S5b) does not confirm any compositional changes of the material. These findings indicate that the material degradation results in an amorphous layer, which possibly protects the underneath perovskite material from complete decomposition. The problem can be solved if the particles are stored and handled under strict exclusion of moisture. Figure 5e,f shows that there are no changes after 48 and 96 h, respectively. It can be concluded that the samples are stable and with that, examination of the pure crystal facets is possible.

### 3.2. Ensemble Measurements of Optoelectronic Properties

**Figure 6:**
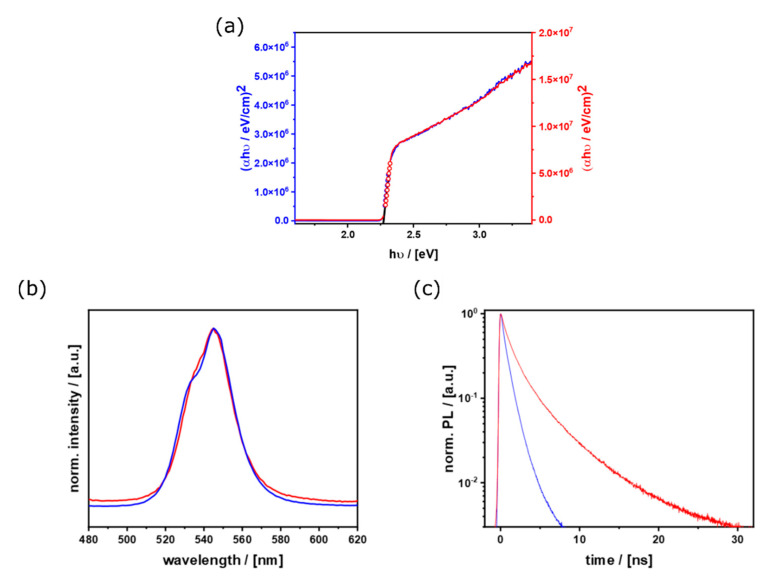
Results from ensemble measurements: (**a**) Tauc plots from UV/Vis absorption data for CU (red) and RD (blue) particles fitted and calculated bandgap of 2.27 eV for both morphologies; (**b**) PL emission spectra and (**c**) time resolved PL decays for both morphologies (blue: RD, red: CU).

We have produced two types of hybrid perovskite microcrystals that differ significantly in their shape and in the crystal facets that terminate the crystal. Since the RD and CU microcrystals have a similar particle size distribution and thus a similar surface-to-volume ratio, they are ideally suited for direct comparison. Figure 6a shows the optical absorption spectrum of the samples and the resulting Tauc-plots. The band-gap is 2.27 eV for both morphologies. This value is in perfect agreement with the literature on MAPbBr_3_ microcrystals [66]. The morphology has no influence on the absorption properties. The different intensities are caused by a difference in the density of microcrystals on the substrate for RD and CU, and is not caused by a change in the absorption coefficient (Figure S6a–c) [67]. The absolute number of particles collected on the substrates is always connected to statistics and cannot be controlled completely by the synthetic parameters. These findings confirm our expectation that the optical absorption property is a bulk-property, which should not depend on the particle morphology.

The situation is different when considering the properties of photoluminescence (PL), as the photogenerated charge carriers (e^−^, h^+^) can be generated either in the bulk or at the surface of the material [38]. They also tend to diffuse to interfaces where they can be trapped before radiative recombination [43,48,49]. Therefore, the nature of particle surfaces should be important. Figure 6b shows the PL spectra. Apparently, there is a small blue shift when comparing RD and CU microcrystals. However, the signal is composed of a double-peak structure as described by Schoetz et al. [68]. The intensity of the λ_em_ = 534 nm signal is obviously weaker than at *λ*_em_ = 544 nm for CU as for RD-microcrystals. The different intensity ratio of the two peaks for the two morphologies can be explained by stronger or weaker self-filtering and self-absorption effects [66,68]. These effects can be caused by the 45° geometry for excitation and detection, in combination with the geometry of the particles. The CU particles show a larger side facet, perpendicular to substrate and top-facet. Thereby the absorption of this facet can be enhanced and extends the charge carriers way through the material (Figure S7), causing a more pronounced red emission than for the RD particles. The time-resolved PL (tr-PL) measurements are given in Figure 6c. The lifetime of the excited species is significantly prolonged when (100) surfaces are present. For the (100) facet, the lifetime is about two times as high as for the (110) facet (Figure S8). When comparing different particle sizes for the same morphology (Figure S9), no significant difference of the curves can be observed. From that and the comparable size of CU and RD particles (Figure S5b), it can be concluded, that the reason for the different lifetimes of the compared morphologies (Figure 6c) is caused by the different crystal facets. The longer lifetime for (100) surfaces can be explained by the more effective separation of h^+^ and e^−^ in CU compared to RD microcrystals. This could also be a consequence of the different surface energy and surface potential of (100) compared to the (110) surfaces. To test the latter hypothesis, ab-initio calculations using the BAND code were performed.

### 3.3. Ab-Initio Calculations and Theoretical Considerations

**Figure 7:**
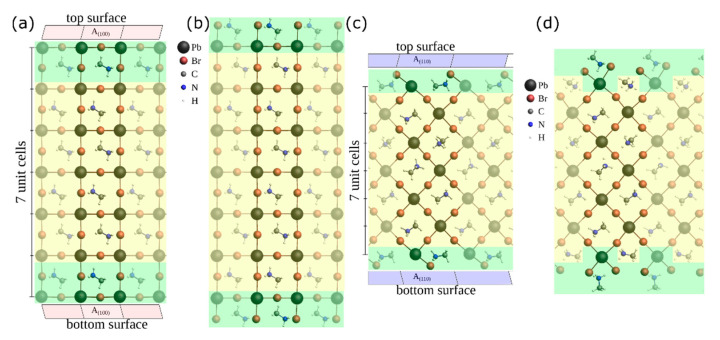
Four types of slabs with lateral periodic boundary conditions (pbc) with (100)-surfaces on the left and (110)-surfaces on the right (Transparent atoms indicating the periodically continued supercells; view along the y-axis). The surface region is marked with a green background and the inner region with a yellow background. The (100)-surface can be terminated by a (**a**) ${\mathrm{PbBr}}_{2}$-layer or by a (**b**) MABr-layer. The (110)-surface can be terminated by a (**c**) mixed layer or by (**d**) MABr-molecules which lead to a two-fold excess of MABr. The (100)-slabs have an area $A_{\left(100\right)}=35.105\;{Å}^{2}$ and the (110)-slabs have an area of $A_{\left(110\right)}=49.649\;{Å}^{2}$ each on the top and on the bottom surface.

To understand the micro single crystals in terms of chemical bonding, the surfaces are studied by ab-initio calculations in a slab model, as explained for example by Reuter [69]. Based on experimental single-crystal X-ray powder diffraction measurements of the bulk perovskite at room temperature (with lattice constant of a=5.9328 Å) [62], bulk-like slabs can be generated as illustrated in Figure 7 suitable for a theoretical approach. The orientation of the MA cation is highly dynamic at room temperature [70]. We have chosen a paraelectric orientation of the MA cation.

The slabs can vary in size, surface orientation, and surface termination, among other things. However, the size has to be limited due to expensive computational resources required for ab-initio calculations, especially because we have to take spin-orbit coupling within ZORA approximation into account [71]. Since we want to get an idea about the stability of the (110)- and (100)-surfaces with several surface terminations we have to set up a suitable slab model and have to compromise between slab size, basis set, and Monkhorst-Pack k-space grid as explained in detail below. To address all these questions in an approximative manner we have chosen the slab model described below. It is obvious that with a larger base set and a larger k-grid a higher accuracy can be achieved, but this is associated with a much higher computational effort.

Since we are interested in understanding the perovskite microcrystals, we focused on surfaces with (100)- and (110)-facets. For both, we construct two different structures with a surface termination by either MABr or ${\mathrm{PbBr}}_{2}$ excess. All four possibilities are shown in Figure 7 for a slab model with seven unit cells. For the (100)-surface, an excess of MABr or ${\mathrm{PbBr}}_{2}$ at the surface is possible so that the slab is terminated either by a MABr layer (a) with an excess number of ${\mathrm{PbBr}}_{2}$ with $N_{\mathrm{excess}}\left({\mathrm{PbBr}}_{2}\right)=-1$ or a ${\mathrm{PbBr}}_{2}$ layer (b) with $N_{\mathrm{excess}}\left({\mathrm{PbBr}}_{2}\right)=1$ respectively. In contrast, for the (110)-direction, it is only possible to obtain either a slab with a two-fold excess of MABr (d) with $N_{\mathrm{excess}}\left({\mathrm{PbBr}}_{2}\right)=2$ or without an excess of either component (c) with $N_{\mathrm{excess}}\left({\mathrm{PbBr}}_{2}\right)=0$ to obtain a charged balanced ionic structure. So, the surface of the latter one consists of a mix of MABr and ${\mathrm{PbBr}}_{2}$.

To understand these different surface compositions from a chemical point of view, a perovskite crystallite could be imagined that forms in the gas phase from ${\mathrm{PbBr}}_{2}\left(g\right)$ and MABr(g) species

(2) $$\mathrm{MABr}(g)+{\mathrm{PbBr}}_{2}(g)\;\to \;\mathrm{MAPb}{\mathrm{Br}}_{3}(s).$$

When chemical equilibrium is reached, a certain surface termination is established related to the partial pressures of the species. Theoretically the surface tension γ can be calculated by dividing the grand canonical potential Ω by the surface area [72]. A similar grand canonical approach has been used by Huang et. al. where they calculated the grand canonical potential Ω of the ${\mathrm{MAPbBr}}_{3}$ (100) surface dependent on the chemical potentials of gaseous ${\mathrm{Br}}_{2}$ and solid Pb with respect to certain reference states [73].

Here we use MABr and ${\mathrm{PbBr}}_{2}$ as independent chemical components in the first step and in the second step we can use the chemical equilibrium of Equation (2) to eliminate the chemical potential of MABr. In the discussion, we are then left with an independent chemical potential of ${\mathrm{PbBr}}_{2}$, which will suffice for an initial exploration of the problem. The surface tension γ can then be approximated as

(3) $$\gamma =\frac{\Omega }{2A}\approx \frac{1}{2A}\left[E_{\mathrm{slab}}\left({\mathrm{MAPbBr}}_{3}\right)-N\left(\mathrm{bulk}\right)E_{\mathrm{bulk}}\left({\mathrm{MAPbBr}}_{3}\right)-N_{\mathrm{excess}}\left({\mathrm{PbBr}}_{2}\right)\mu \left({\mathrm{PbBr}}_{2}\right)\right]$$

Here $E_{\mathrm{slab}}\left({\mathrm{MAPbBr}}_{3}\right)$ refers to the total energy of the ab-initio calculated perovskite slab, $E_{\mathrm{bulk}}\left({\mathrm{MAPbBr}}_{3}\right)$ is the total energy of a bulk perovskite cell, N(bulk) the number of complete ${\mathrm{MAPbBr}}_{3}$ units in the slab, and A is the area of the top and bottom surface of our slab as shown in Figure 7. The formula shows the dependence of the surface tension on the chemical potential $\mu \left({\mathrm{PbBr}}_{2}\right)$ of ${\mathrm{PbBr}}_{2}$ and the excess of this component in the surface $N_{\mathrm{excess}}\left({\mathrm{PbBr}}_{2}\right)$ in accordance with the well-known Gibbs-adsorption isotherm [72].

The surface tension can thus theoretically be influenced by tuning the chemical potential with respect to a suitable reference state. Here we can use the chemical potential of solid ${\mathrm{PbBr}}_{2}$, hence, we treat the hypothetical case where solid perovskite and solid ${\mathrm{PbBr}}_{2}$ are present side by side in chemical equilibrium and $\mu \left({\mathrm{PbBr}}_{2}\left(g\right)\right)$ is equal to $\mu \left({\mathrm{PbBr}}_{2}\left(s\right)\right)$. In this case, we would formally consider the saturation pressure of $p\left({\mathrm{PbBr}}_{2}\left(g\right)\right)=p{\left({\mathrm{PbBr}}_{2}\left(g\right)\right)}^{\mathrm{sat}}$ of the solid ${\mathrm{PbBr}}_{2}$ in the gas phase.

For this reference state, we calculate the surface tensions of the (100)-surface and the (110)-surface i.e., for the slab models shown in Figure 7.

Ab-initio calculations with the BAND code implemented in the SCM (Software of Chemistry and Materials B.V.) suite on PBE (Perdew-Burke-Ernzerhof) level of theory with spin-orbit coupling and a double zeta basis set (DZP) were performed [74,75,76]. Lattice optimization of the bulk perovskite cell provided a lattice constant value a=5.925Å which is nearly identical with the experimental value given above.

To stay close to the cubic bulk structure in the inner cell region (Figure 7, marked in yellow), our slab model constrains the Br−Pb−Br angles and the Pb−Pb−Pb angles to 90° during geometry optimization (until the Hellmann-Feynmann forces are below 27 meV/Angstrom for each atom). These restrictions still allow the inner cells to expand or compress, while the atoms at the surface region (Figure 7, marked in green) could relax without constraints.

A Monkhorst-Pack k-space grid of 5 × 5 × 3 is used for the total energy calculations of the geometry optimized slab model. With total energy calculations of relaxed perovskite slabs, bulk perovskite cells, and bulk ${\mathrm{PbBr}}_{2}\left(s\right)$, the surface tension can be calculated via Equation (3).

The results are given in Table 1. It should be noted that the entire discussion is based on single-point calculations of geometry-optimized structures reflecting a formal situation at *T* = 0 K. However, this does not take into account the vibrational zero point energies, excess entropies, and excess heat capacities of the surfaces that have been studied for simpler surfaces such as MgO(110) [77]. Note that the mass equilibrium at *T* = 0 K is orthorhombic and not cubic [70]. The ab-initio calculations at *T* = 0 K therefore represent a pseudo cubic structure and provide a crude model of the average cubic perovskite at room temperature. Moreover, extrapolations to infinite slab sizes seem to be out of range for the perovskite slab model.

Within our slab model, the values for the (100)-surface are higher than the values calculated by Huang et al. for their reference states. It should be observed, that in their theoretical slab model they use an optimized lattice constant of a=6.08 Å larger than the experimental value of a=5.9328 Å and our optimized value of a=5.925 Å with a plane-wave basis approach within VASP-code with a k-grid of 8 × 8 × 1 while in our case a k-grid of 5 × 5 × 3 was chosen within BAND-code. They also used the restriction that the C–N bond of the MA cations is in the (111)-direction to stay close to the cubic bulk structure, which is different to our approach.

The extraction of the gamma values by use of Equation (3) is a delicate task. However, as will be shown below, our interest lies not in the absolute surface tensions, but in the ratios, which can be discussed in a consistent manner with the chosen model.

According to Table 1, in our reference state the ${\mathrm{PbBr}}_{2}$-terminated surface is slightly more stable than the MABr terminated (100)-surface. The values of the (110)-surfaces are only slightly higher than the (100)-surfaces by about 0.1 J/m². The ratios $r=\gamma_{\left(110\right)}/\gamma_{\left(100\right)}$ lie between 1.23 and 1.26 i.e., $<\sqrt{2}$ hinting a coexistence of (100)- and (110)-surface type facets for cubic microcrystals for this reference state according to the usual Wulff construction which is explained in detail e.g., by Lupis et al. [72].

The crystallite shape is thereby geometrically characterized by the distances $h_{\left(110\right)}$ and $h_{\left(100\right)}$ from their corresponding (110)- and (100)-facets to the crystallite center. In thermodynamic equilibrium, the surface tension ratios determine the h-ratio according to $h_{\left(110\right)}/h_{\left(100\right)}=\gamma_{\left(110\right)}/\gamma_{\left(100\right)}$.

**Figure 8:**
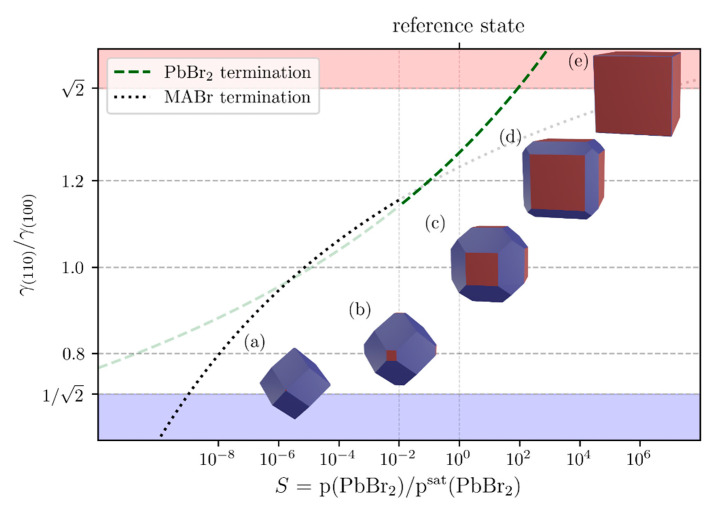
Dependence of the quotient r of the surface tensions for (110) and (100)-surfaces on the saturation number S. This is shown for the ${\mathrm{PbBr}}_{2}$-terminated surfaces (Figure 7a,c) (green dashed lines) and for the MABr-terminated surfaces (Figure 7b,d) (black dotted lines). Since the MABr termination below $S=1.0\cdot 10^{-2}$ is thermodynamically more stable than the ${\mathrm{PbBr}}_{2}$ termination, the green dashed curve below this value and the black dotted curve above this value are drawn transparently. For five quotients, 3D-Wulff constructions of cubic crystallites (**a**–**e**) with (100) (red) and (110)-surfaces (blue) were shown, with the (100)-surface becoming more and more dominant for higher $\gamma_{\left(110\right)}/\gamma_{\left(100\right)}$-values. For geometric reasons, below a quotient of $r=1/\sqrt{2}$, i.e., in the blue region, the (100)-facet and above a quotient of $r=\sqrt{2}$, i.e., in the red region, the (110)-facet vanish.

The corresponding crystallite shape for our reference state at $\mu_{\mathrm{ref}}=\mu \left({\mathrm{PbBr}}_{2}\left(g\right)\right)=\mu \left({\mathrm{PbBr}}_{2}\left(s\right)\right)$ i.e., with $p\left({\mathrm{PbBr}}_{2}\right)=p{\left({\mathrm{PbBr}}_{2}\right)}^{\mathrm{sat}}$ is shown in Figure 8b, for example temperature T of 500 K. Theoretically, there could be additionally a (111)-surface which is not investigated in this work since it had not been observed in the experiment.

Figure 8 shows furthermore how the equilibrium shape varies with the partial pressure of $p\left({\mathrm{PbBr}}_{2}\right)$ with respect to the saturation pressure $p{\left({\mathrm{PbBr}}_{2}\right)}^{\mathrm{sat}}$ and hence with the saturation number $S=p\left({\mathrm{PbBr}}_{2}\right)/p{\left({\mathrm{PbBr}}_{2}\right)}^{\mathrm{sat}}$ via the pressure dependence of the chemical potential $\mu \left({\mathrm{PbBr}}_{2}\left(g\right)\right)=\mu \left({\mathrm{PbBr}}_{2}\left(s\right)\right)+k_{B}T\ln\left(p\left({\mathrm{PbBr}}_{2}\right)/p{\left({\mathrm{PbBr}}_{2}\right)}^{\mathrm{sat}}\right)$ and accordingly to Equation (3).

Saturation values S<1 corresponds to $p\left({\mathrm{PbBr}}_{2}\right)<p{\left({\mathrm{PbBr}}_{2}\right)}^{\mathrm{sat}}$. Values S>1 represent supersaturations of ${\mathrm{PbBr}}_{2}$ gas with respect to nucleation of ${\mathrm{PbBr}}_{2}$ solid.

By varying the saturation number, which influences each surface tension differently due to $N_{\mathrm{excess}}\left({\mathrm{PbBr}}_{2}\right)$, one can get the shape of the microcrystals. 

If the ratio $r=\gamma_{\left(110\right)}/\gamma_{\left(100\right)}$ is 1 none of the surfaces is thermodynamically favored resulting in a crystallite shape shown in Figure 8, polymorph (a). With an increase in r the (110)-surface becomes less and less thermodynamically favored resulting in the ECC shaped particles, showing (100)- and (110)-surfaces (Figure 8, polymorph (c)). Due to Wulffs construction, there are only (100)-surfaces left (Figure 8, polymorph (e)), if r becomes larger than $\sqrt{2}$ resulting in CU microcrystals.

Because of the different trends in surface tension of the terminated surfaces based on $N_{\mathrm{excess}}\left({\mathrm{PbBr}}_{2}\right)$, the thermodynamically more stable termination discussed in Table 1 changes from the ${\mathrm{PbBr}}_{2}$-terminated surface to the MABr-terminated surface at a saturation factor of about $1.0\times 10^{-2}$ (Figure 8).

Despite the crudeness of the thermodynamic slab model, it nevertheless provides the basis for the discussion of a variety of shapes in terms of equilibrium states in good agreement with the herein reported experimental observations. As usual, such equilibrium states can provide a solid basis for further dynamic processes between these states and shapes i.e., the reaction dynamics of crystal formation.

This discussion of shapes can be in principle analogously transferred to formation in solution by calculating the saturation S as a ratio of concentrations or more correctly activities. However, theoretically solid/solvent interfaces would have to be simulated within the slab model in a grand canonical ensemble which is a very difficult task.

Besides all of this, we have to state that our discussion has been based on relatively small slab structures. Our discussion is also based on the equilibrium of Equation (2). A more realistic analysis must be based on independent components ${\mathrm{PbBr}}_{2}$ and MABr.

### 3.4. Single-Particle Measurements

As already mentioned above, the herein reported aerosol synthesis was optimized to offer the possibility to synthesize microcrystals with very clean surfaces. Therefore, the surfaces are smooth enough to directly characterize them using local methods like KPFM. The orientation and distribution of the particles gives the opportunity to determine facet, and with that direction, selective electronic properties of the perovskite material. The theoretically determined difference in the surface tension of the two different facets is expected to be measurable for the synthesized microcrystals.

**Figure 9:**
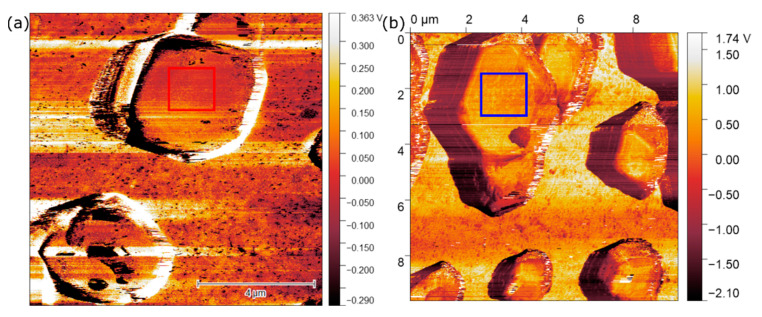
KPFM images for (**a**) (100) and (**b**) (110) facets from CU, respectively RD particles. From the red and blue marked area an average CPD of 0.389 V for (100) and 0.349 V for (110) is determined. The amplitude images are added in Figure S10.

The contact potential difference (CPD) maps received from the KPFM measurements are shown in Figure 9. In order to avoid effects of topography cross-talk in the interpretation, we chose the crystal surfaces that were parallel to the substrate (marked areas) to obtain CPD values and for work function calculation. The CPD distribution was uniform on both crystals, suggesting absence of band-bending or huge defect density gradient on the surfaces [78]. The work functions were calculated according to Equation (1) as 4.864 ± 0.429 eV for the (100) and 4.661 ± 0.223 eV for the (110) surface. The higher working function for the (100) facet means a higher energy is needed to release an electron from this surface. This leads to the assumption that the electronic properties show dependency on the crystal orientation.

The micrometer-sized, ligand-free crystal surfaces allow direct comparison of the photophysical properties of the differently oriented facets. When optically investigating micro structured materials, care needs to be taken to differentiate between bulk and surface properties, size-dependent effects as well as distortions and artefacts arising on purely geometric and ray optical grounds. In MAPbBr_3_ crystals, photoluminescence (PL) spectra have been found to exhibit dependence on particle size [66], double-emission attributed to self-absorption [68], as well as the location of the excitation at the surface vs. in the bulk of the crystal [40].

**Figure 10:**
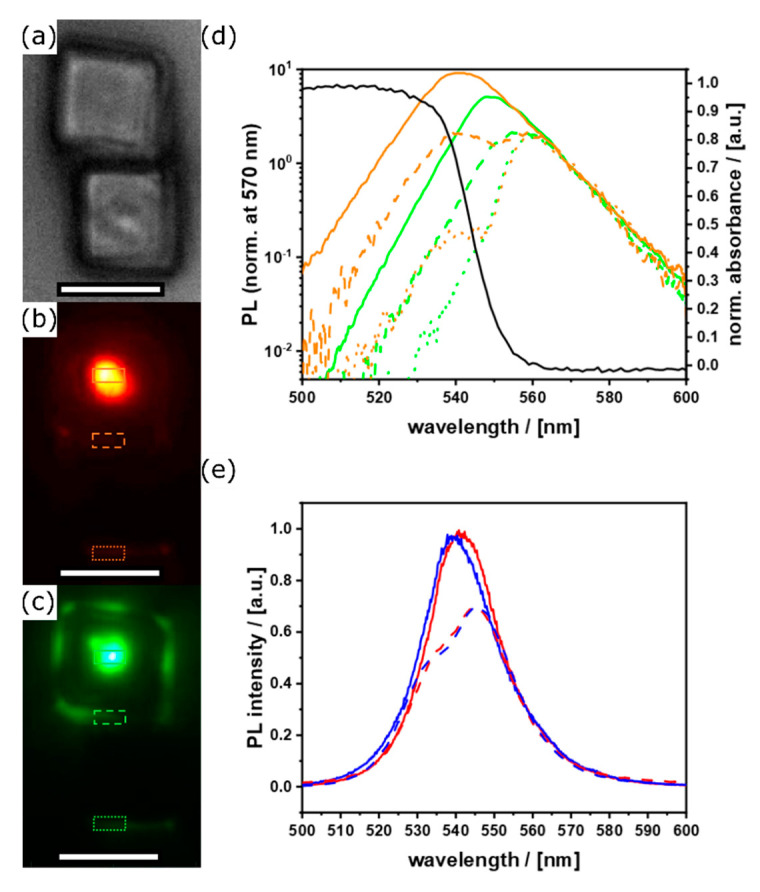
(**a**) Optical brightfield micrograph (scale bars = 10 μm) of neighboring cubes, false color representations of PL emission generated by (**a**) linear absorption at 400 nm and (**c**) 2PA at 800 nm; (**d**) corresponding emission spectra at the positions as indicated by the colored boxes in (**b**) (orange lines) and (**c**) (green lines), absorbance spectrum of the same sample (black line). (**e**) Comparison of µPL (solid lines) and ensemble measurements (dashed lines) of RD (blue) and CU (red) particles.

By both resolving the emission with an imaging spectrograph, and selectively exciting only a small, controlled volume of each particle, we are able to disentangle the above effects and obtain truly facet-selective spectra. When detecting PL away from the excitation spot, the higher energy shoulder of the peak is suppressed as emitted light is partially reabsorbed due to the small Stokes shift inherent to hybrid perovskite materials, resulting in an asymmetric peak with a red-shifted emission maximum. This effect becomes more pronounced with increasing excitation detection distance, and was even observed to occur through neighboring crystals (Figure 10a), where one particle effectively acts on the other’s emission as an optical long-pass filter. Generating the photoexcitation in the bulk of the crystal by means of two-photon absorption (2PA) (Figure 10c), in contrast to predominantly at or near the surface as is the case in linear absorption (Figure 10b), provides a potent way of testing and verifying these results.

Comparing the emission spectra collected directly at the central excitation spot, at the edge of the excited crystal, and the far edge of the neighboring crystal, the increasing self-filtering effect becomes apparent (Figure 10d). In the case of linear absorption, this results in an additional shoulder on the red side of the spectrum, in addition to the initial peak centered at 540 nm. Ensemble PL measurements (Figure 10b,c), using a larger excitation spot size and a 45° geometry for excitation and detection, are subjected to this artefact, since illumination of the particle sides are detected at a total of 90°. Thus, a portion of the detected light has to traverse some material on the way to the detector, hence yielding self-absorption distorted contribution to the spectra (Figure S7). Figure 10e demonstrates this behavior by comparing the ensemble measurements collected orthogonally to the excitation beam to the selective, antiparallel top facet excitation on individual crystals. The data presented as the solid lines were selectively collected at the center portion of the differently oriented facets only, after excitation at 400 nm, yielding artefact-free surface emission spectra directly correlated to the corresponding lattice terminations. We note a slight blueshift of the high-energy side for the 110 direction, as well as an accentuated broadening of the peak toward the red for the 100 direction. These features, although small, occur uniformly for all measured particles for each geometry (Figure S11).

**Figure 11:**
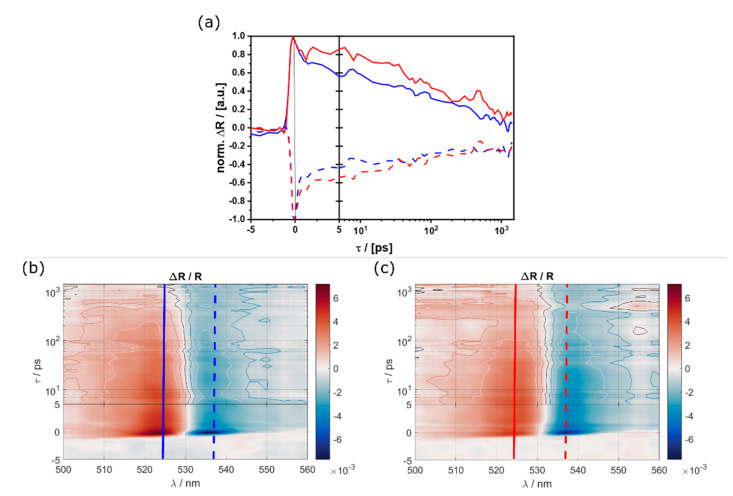
(**a**) Differential reflection transients at 524 nm (solid lines) and 537 nm (dashed lines) for CU (red) and RD (blue) particles; and full transient reflection maps measured on individual (**b**) (110) and (**c**) (100) facets, vertical lines corresponding to (**a**), contour lines at longer delay times indicate steps of 10^−3^.

As shown before (Figure 6c), time-resolved PL traces were collected from the various morphologies by means of time-correlated single photon counting, using 405 nm excitation in order to predominantly probe the surface states at the different terminations. We find the (100)-facets to exhibit significantly longer lifetimes compared to the (110), in accordance with previous results [43]. Extending our studies to shorter timescales, local transient reflection measurements were performed, with a broadband probe pulse focused on either the (100) or the (110) top facet of a single microcrystal following a defocused 400 nm pump pulse (Figure 11). An ultrafast decay component in the order of 1ps is more pronounced for the 110 terminated surfaces, while a slower component is similar for both cases (Figure 11a). Furthermore, the zero-crossing of the transient reflection spectra, indicated by the black contour line in Figure 11a, is blue shifted by a few nm for the 110 facets. This is related to the real part of the complex dielectric constant via the Kramers-Kronig relations and indicates a shift of the renormalized bandgap following excited charge carrier generation, which is consistent with the blueshift observed in the µPL spectra discussed above.

With regard to future applications of the microcrystals presented in this work, lasing is of particular interest. Several examples can be found in the literature, e.g., MAPbBr_3_ nanowires or all inorganic CsPbBr_3_ microcubes [79,80]. In the context of the previously mentioned surface- and shape-dependent properties of the materials, another interesting application could be shape-dependent lasing, which was mentioned by Zhang et al. for MAPbBr_3_ microstructures [81]. Another report about the size dependency of the superluminescence threshold in MAPbBr_3_ microcubes can be found [66]. Furthermore, lasing from Mie-Resonant CsPbBr_3_ nanocubes has been recently reported, offering another possibility for application [82]. All the above mentioned examples illustrate the importance of microcrystals for such applications and their great potential for optoelectronic devices.

## 4. Conclusions

The presented work demonstrates the advantages of aerosol-processed perovskite microcrystals as a model system for a better understanding of shape-related properties. By optimizing the liquid precursor for an aerosol-assisted synthetic route and the synthesis setup, we were able to precipitate defined microcrystals with pure surfaces. The separation of particles from liquid residues is important to suppress post-synthetic processes, such as Ostwald ripening, and other influences by impurities, e.g., liquid precursor remains. With small changes in the composition of the liquid single-source precursor, without using capping agents, we were able to control the shape of the particles. This offers the possibility to create defined and oriented MAPbBr_3_ microcrystals on different substrates and to determine their facet-selective optoelectronic properties. It could be seen that the obtained micrometer sized single crystals are stable under dry conditions, enabling the characterization of defined and pristine single crystal facets, not being influenced by any size-dependent effects. Using spatially resolved UV/Vis spectroscopy, we were able to show the emission of the (100) surface is slightly red shifted compared to the (110) facet. By applying a broad variety of methods, including 2PA and comparing spatially resolved single particle and particle ensemble measurements, we can exclude previously reported effects, like self-absorption and self-filtering, as the cause of this red shift. Time-resolved PL measurements confirmed the previously reported extended charge carrier lifetimes for the (100) facets. This can be explained by the higher stability of this surface and the associated lower defect density compared with the (110) facet. The higher defect density for (110) facets leads to faster recombination and thus shorter lifetimes. By using KPFM, the work function for the two different facets could be determined, leading to higher values for the thermodynamic (100) facets. Furthermore, the surface tension of both crystal facets was determined by theoretical ab-initio calculations. The simulations resulted in higher surface tensions for (110) facets, which is in full agreement with the experimental results. Moreover, these results allow the thermodynamic explanation of the obtained particle morphologies by using the Wulff construction, relating the surface tensions to the activity of lead bromide. Reducing the activity of the lead bromide species in the liquid precursor, the kinetic barriers are increased, leading to the formation of morphologies dominated by (110) surfaces. The activity of the lead bromide species can be influenced by the addition of MABr and/or DMF, leading to faster crystal formation and with this, dominance of the thermodynamic (100) surfaces. This offers the possibility to adjust the obtained crystal facets and furthermore the optical properties of the obtained perovskite material in a ligand-free synthesis without changing its composition.

## Figures and Tables

**Table 1:** Summary of the surface tensions of the ab-initio calculated structures with (100)- and (110)-surfaces. Slabs with seven unit cells were studied with the four different surface terminations shown in Figure 7. All values are given in J/m². $N_{\mathrm{excess}}\left({\mathrm{PbBr}}_{2}\right)$ values used in Equation (3) are given to mark the excess of ${\mathrm{PbBr}}_{2}$ in the surface region.

Termination	$N_{\mathrm{excess}}\left(PbBr_{2}\right)$	Surface Tension/(J/m²)
(100)-surface		
(a) PbBr_2_	1	0.416
(b) MABr	−1	0.498
(110)-surface		
(c) mixed	0	0.526
(d) 2 × MABr	−2	0.614

## Data Availability

The data presented in this study are contained within the article and the corresponding supplementary material.

## Supplementary Materials

The following are available online at https://www.mdpi.com/article/10.3390/nano11113057/s1, Figure S1: SEM micrographs of microcrystals prepared according to the method reported in reference [43], Figure S2: Schematic representations of PbBr_∞_-chain, Figure S3: Precursor reactivity. UV/Vis studies for different compositions of liquid precursors, Figure S4: Particle size distribution from statistical evaluation of SEM images, Figure S5: Characterization of CU particles under ambient conditions for 6 weeks, Figure S6: SEM overview images of all three morphologies obtained via the aerosol process, Figure S7: Sketch of self absorption in 90° geometry, Figure S8: Time resolved PL spectra (ensemble measurements) fitted by a triexponential fit, Figure S9: Time resolved PL spectra for CU particles with different sizes, Figure S10: Additional data from KPFM measurements, Figure S11: Space resolved PL emission spectra for different facets.
