# Supplementary material for: On the Shape-Selected, Ligand-Free Preparation of Hybrid Perovskite (CH3NH3PbBr3) Microcrystals and Their Suitability as Model-System for Single-Crystal Studies of Optoelectronic Properties
Source: Nanomaterials (Basel). 2021 Nov 13;11(11):3057. doi: 10.3390/nano11113057 (PMC8623308; doi:10.3390/nano11113057)
Supplement: Supplementary file 1 [file nanomaterials-11-03057-s001.zip › nanomaterials-1428682-supplementary.pdf]

nanomaterials

MDPI

Supplementary Materials

On the Shape-Selected, Ligand-Free Preparation of Hybrid Perovskite ($CH_3NH_3PbBr_3$) Microcrystals and Their Suitability as Model-System for Single-Crystal Studies of Optoelectronic Properties

Ulrich Johannes Bahnmüller [1], Henning Kuper [2], Tobias Seewald [3], Yenal Yalçinkaya [4], Jörg August Becker [2], Lukas Schmidt-Mende [3], Stefan A. L. Weber [4,5] and Sebastian Polarz [1,*]

1 Institute of Inorganic Chemistry, Leibniz University Hannover, Callinstraße 9, 30167 Hannover, Germany; ulrich.bahnmueller@aca.uni-hannover.de
2 Institute of Physical Chemistry and Electrochemistry, Leibniz University Hannover, Callinstraße 3A, 30167 Hannover, Germany; henning.kuper@pci.uni-hannover.de (H.K.); Joerg-august.becker@pci.uni-hannover.de (J.A.B.)
3 Department of Physics, University of Konstanz, Universitätsstraße 10, 78457 Konstanz, Germany; tobias.seewald@uni-konstanz.de (T.S.); Lukas.schmidt-mende@uni-konstanz.de (L.S.-M.)
4 Max-Planck-Institute for Polymeric Research, Ackermannweg 10, 55128 Mainz, Germany; yalcinkayay@mpip-mainz.mpg.de (Y.Y.); webers@mpip-mainz.mpg.de (S.A.L.W.)
5 Institute of Physics, Johannes Gutenberg University Mainz, Staudingerweg 7, 55128 Mainz, Germany
* Correspondence: sebastian.polarz@aca.uni-hannover.de

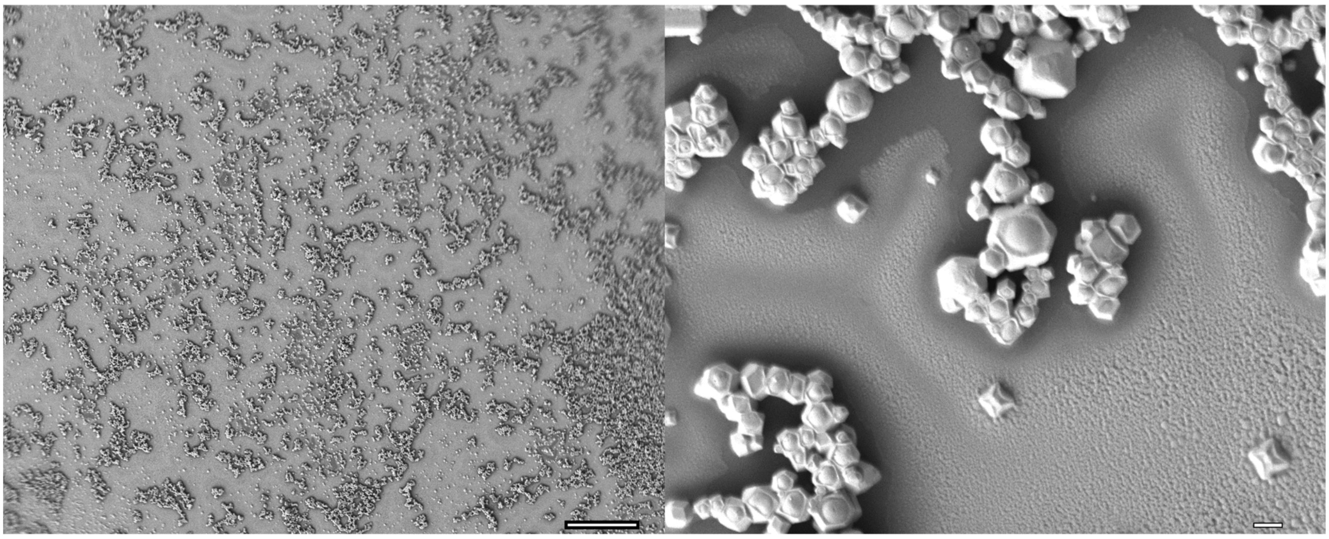

Figure S1. SEM micrographs of microcrystals prepared according to the method reported in reference [43].

a) SEM micrographs (scalebars = 50 µm (left), 1 µm (right)) of $MAPbBr_3$ microcrystals, which are strongly aggregated on the substrate.

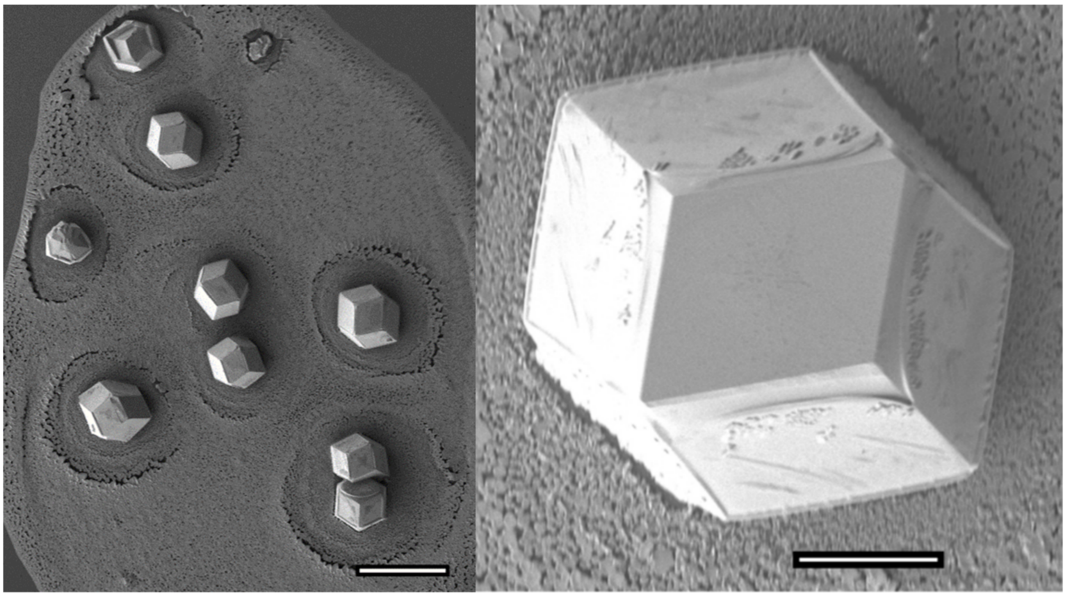

b) SEM micrographs (scalebars = 50 μm (left), 10 μm (right)) of $MAPbBr_3$ microcrystals, which are surrounded by smaller structures.

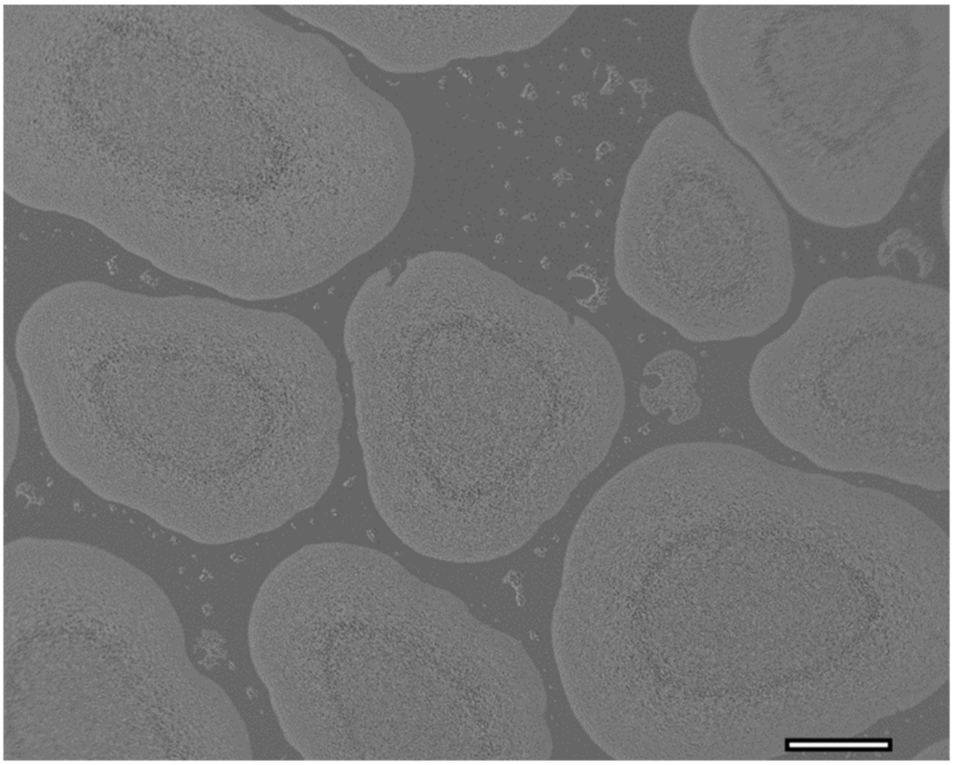

c) SEM micrograph (scalebar = 5 μm), of smaller structures prepared by aerosol processing at lower temperatures.

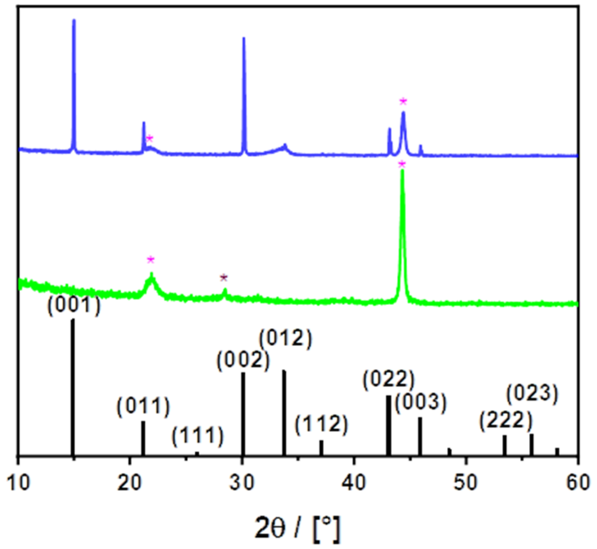

d) PXRD pattern of samples prepared at room temperature (green) and at 150 °C (blue). Reflexes marked with stars belong to the substrate (brown) or the sample holder (pink).

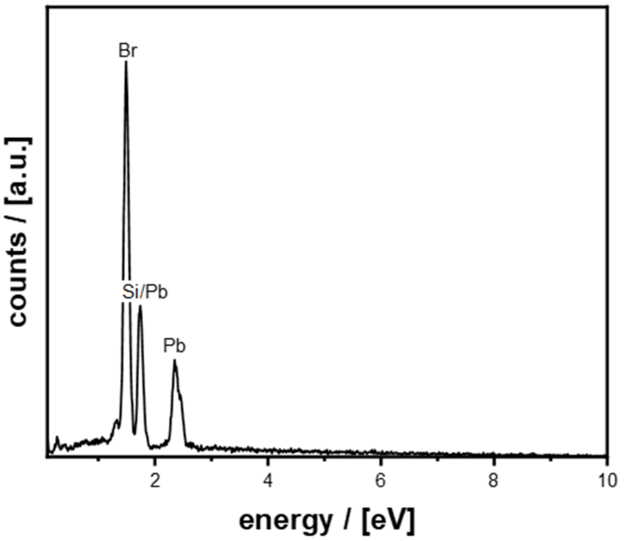

e) EDX spectra of film obtained from synthesis at rt. Si signal is caused by the Si(111)-Wafer used as substrate for particle collection.

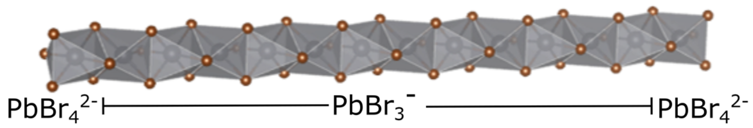

Figure S2. Schematic representation of $PbBr_\infty$-chain.

Exemplary illustration of $PbBr_\infty$ chains of the precursor system with designated $PbBr_4^{2-}$ end groups and $PbBr_3^-$ units building the chains.

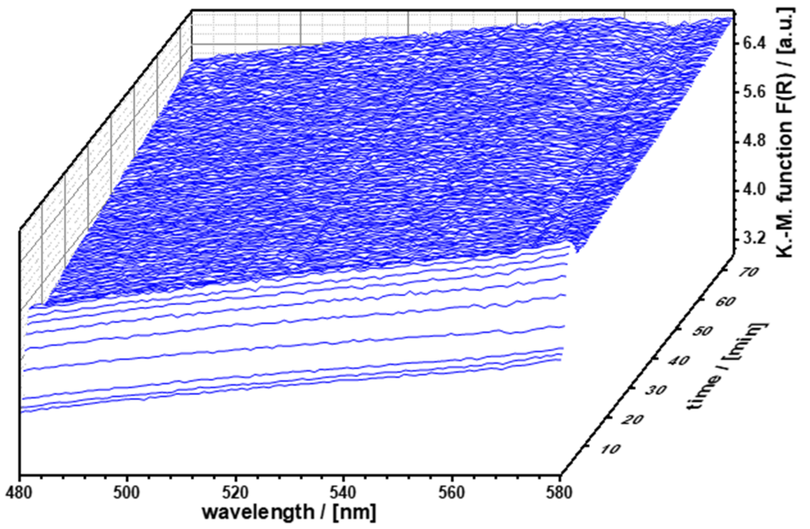

Figure S3. Precursor reactivity. UV/Vis studies for different compositions of liquid precursors.

a) UV/Vis spectra from the conversion of the pure TEG-precursor with equimolar ratios of $PbBr_2$ and MABr. The first measurment made at 35 °C, afterwards temperature was increased to 75 °C with a heating rate of 16 °C/min. There is no conversion from the precursor into the perovskite material observable under the chosen conditions.

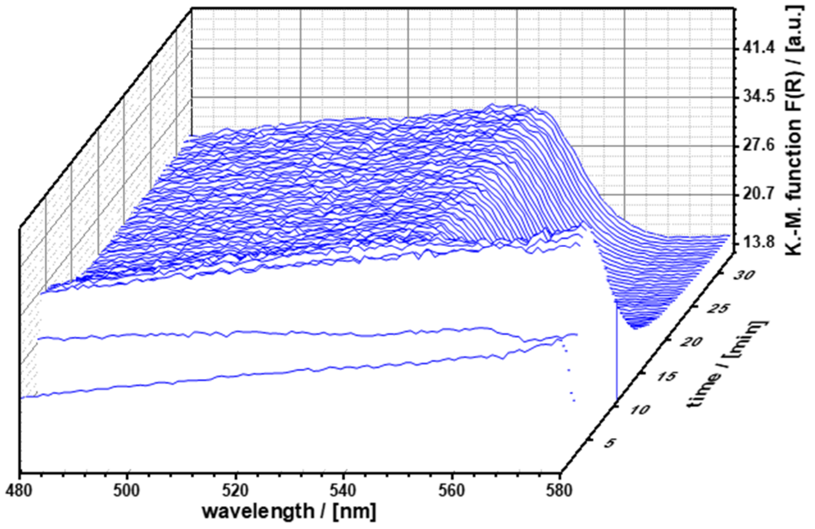

b) UV/Vis spectra (same conditions as before) of the mixed DMF-TEG precursor with equimolar ratios of $PbBr_2$ and MABr. After about 10 min there is a significant change in the Kubelka-Munk function detectable, showing the conversion of the liquid precursor into the perovskite material, showing a band edge.

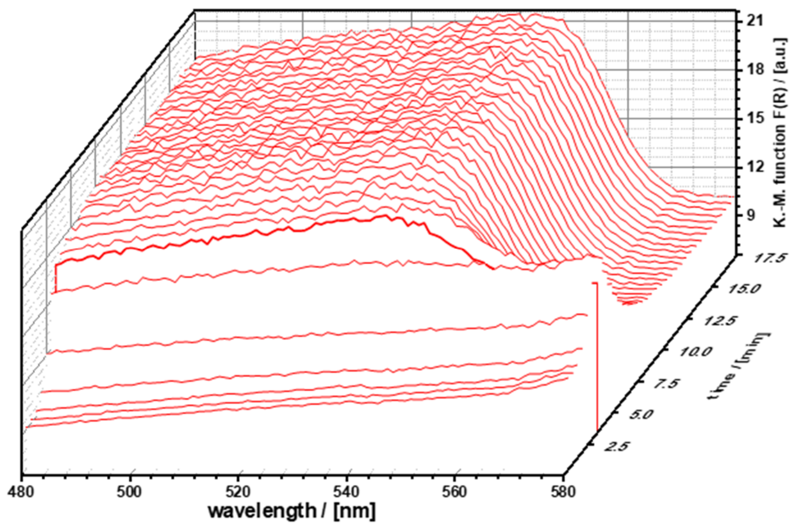

c) UV/Vis spectra (same conditions as before) of the mixed DMF-TEG precursor with 1.8 times excess of MABr. After about 3 min there is a significant change in the Kubelka-Munk function detectable, showing the conversion of the liquid precursor into the perovskite material, showing a band edge.

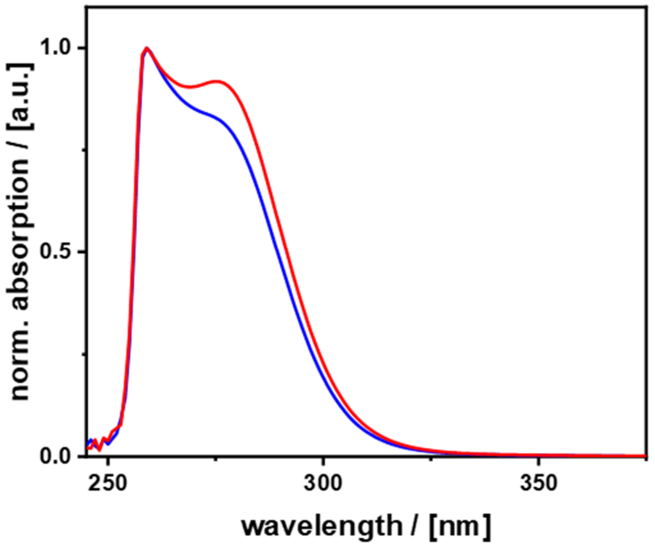

d) UV/Vis spectra of liquid precurors with 1:1 (v:v) TEG to DMF ratio and equimolar ratio of MABr to $PbBr_2$ (red) and excess (1.8 times) MABr (blue).

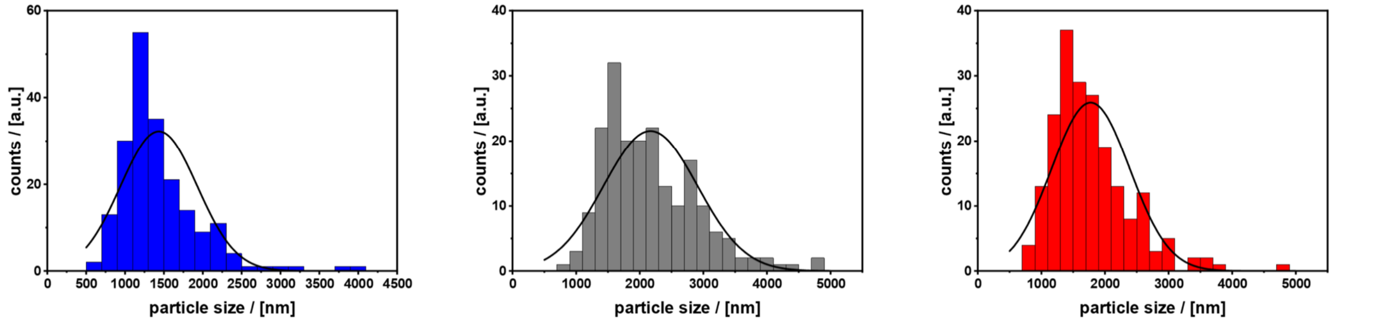

Figure S4. Particle size distribution from statistical evaluation of SEM images.

a) Statistical evaluation of SEM data of RD (blue), ECC (grey) and CU (red) microcrystals. The particle size distribution ranges from several 100 nm to several μm.

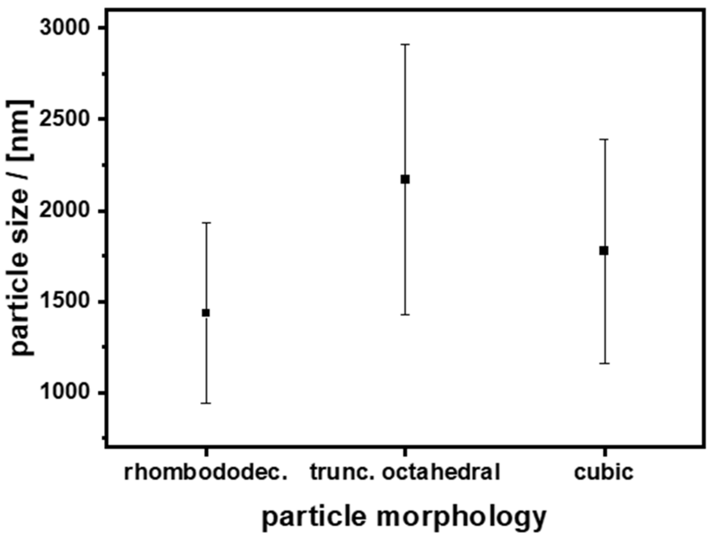

b) Direct comparison of the averaged particle sizes for the three obtained morphologies.

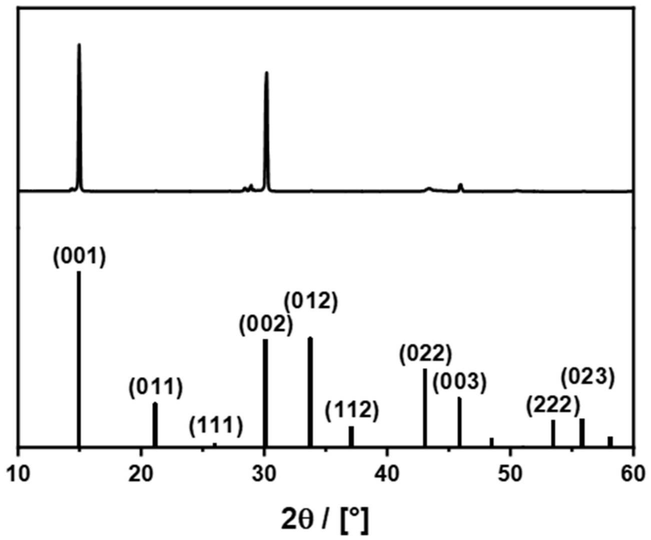

.

Figure S5. Characterization of CU particles stored under ambient conditions for 6 weeks.

a) PXRD pattern of CU sample stored for 42 d under ambient conditions with reference pattern of $MAPbBr_3$. The pattern does not show significant changes, compared to directly measured samples (Figure 4a).

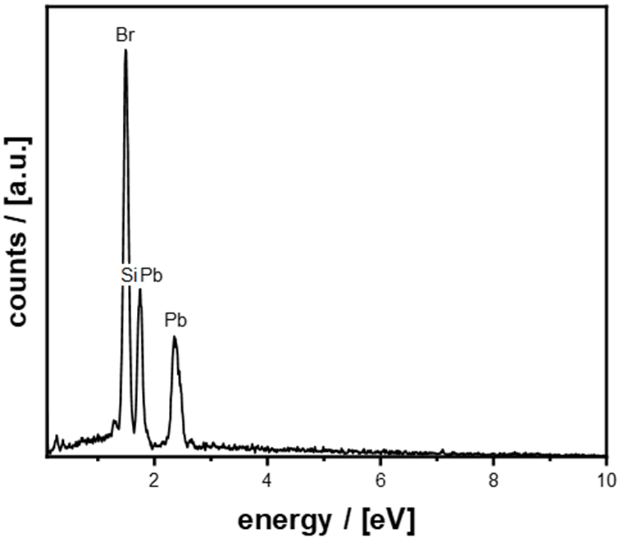

b) EDX spectra of CU particles stored under ambient conditions for 42 d. No deterioration in the composition of the perovskite material is observed.

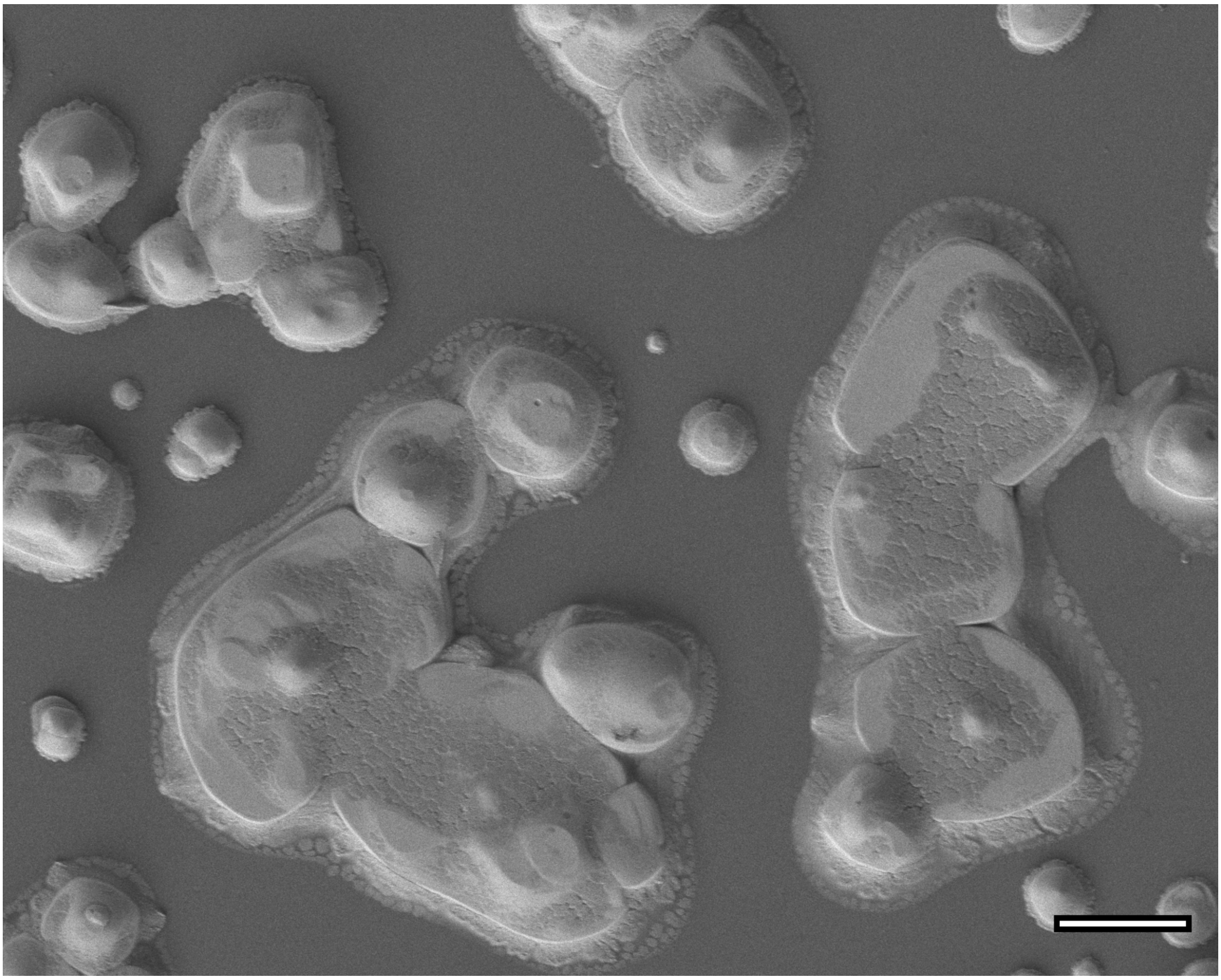

c) SEM image of CU particles stored under ambient conditions for 42 d. Strong morphological decomposition is observed. Almost none particle is left and none particle with defined edges and facets can be found, scale bar = 5 µm.

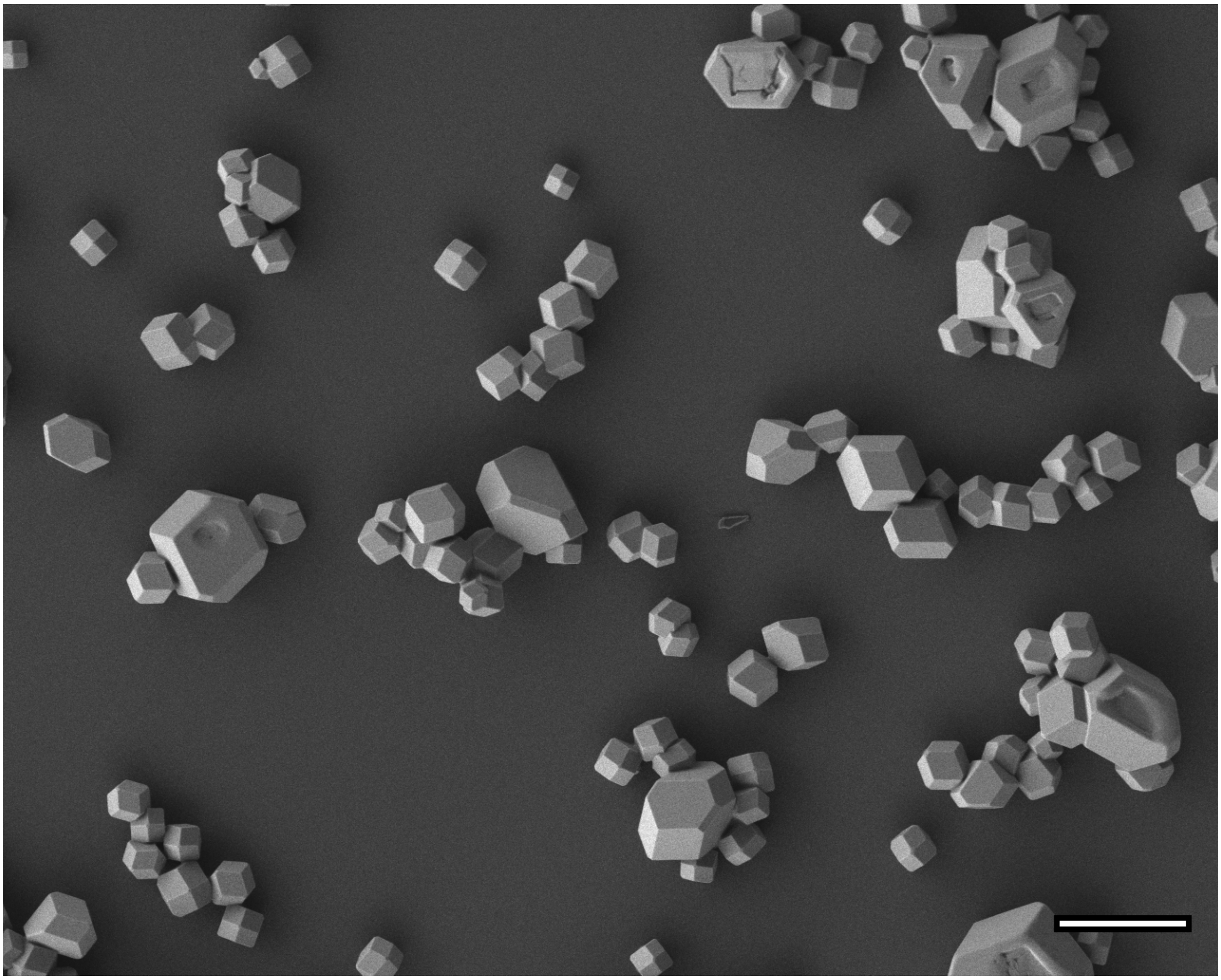

Figure S6. SEM overview images of all three morphologies obtained via the aerosol process.

a) rhombododecahedral particles; scale bar = 5 µm.

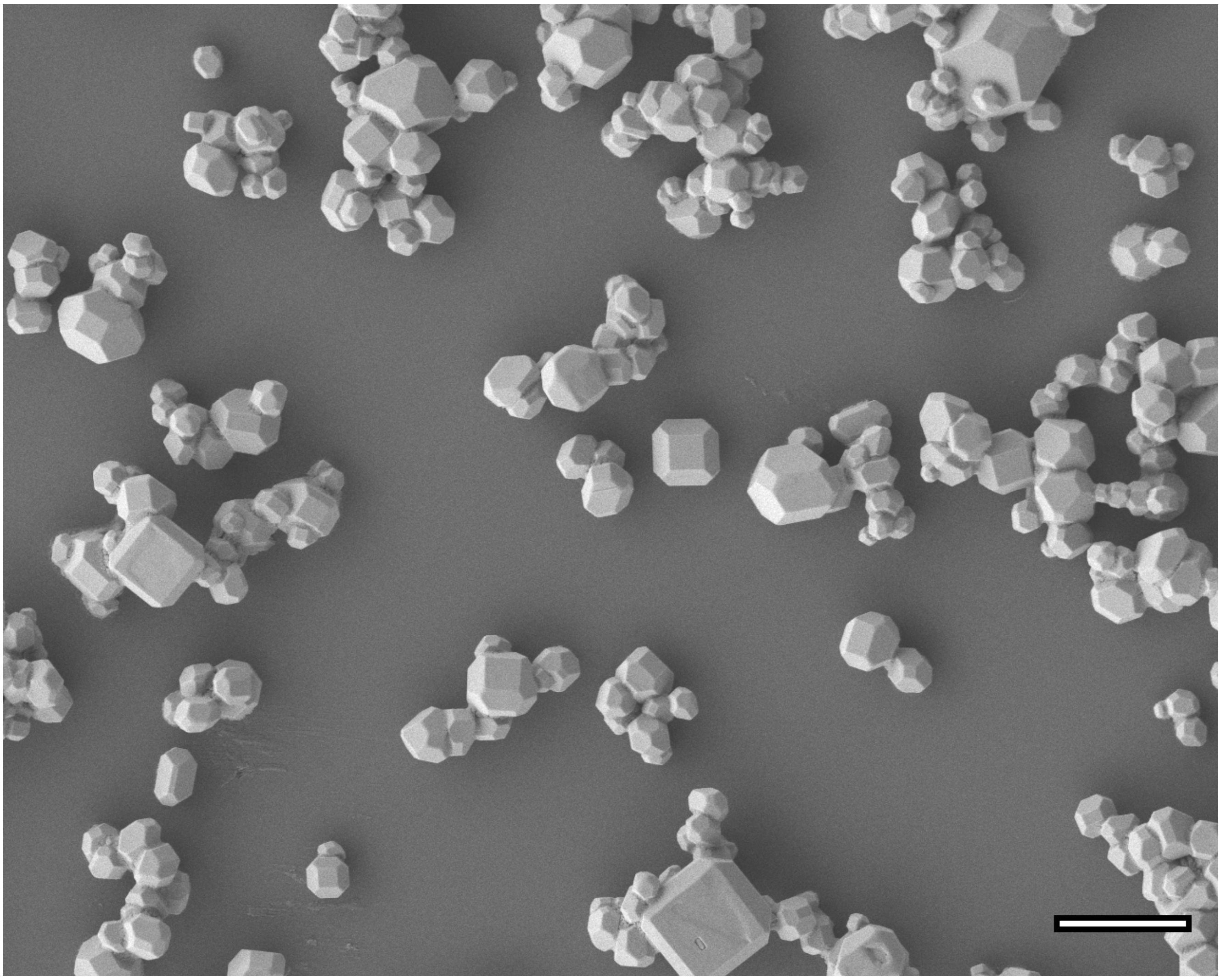

b) edge cut cubic particles, scale bar = 5 μm.

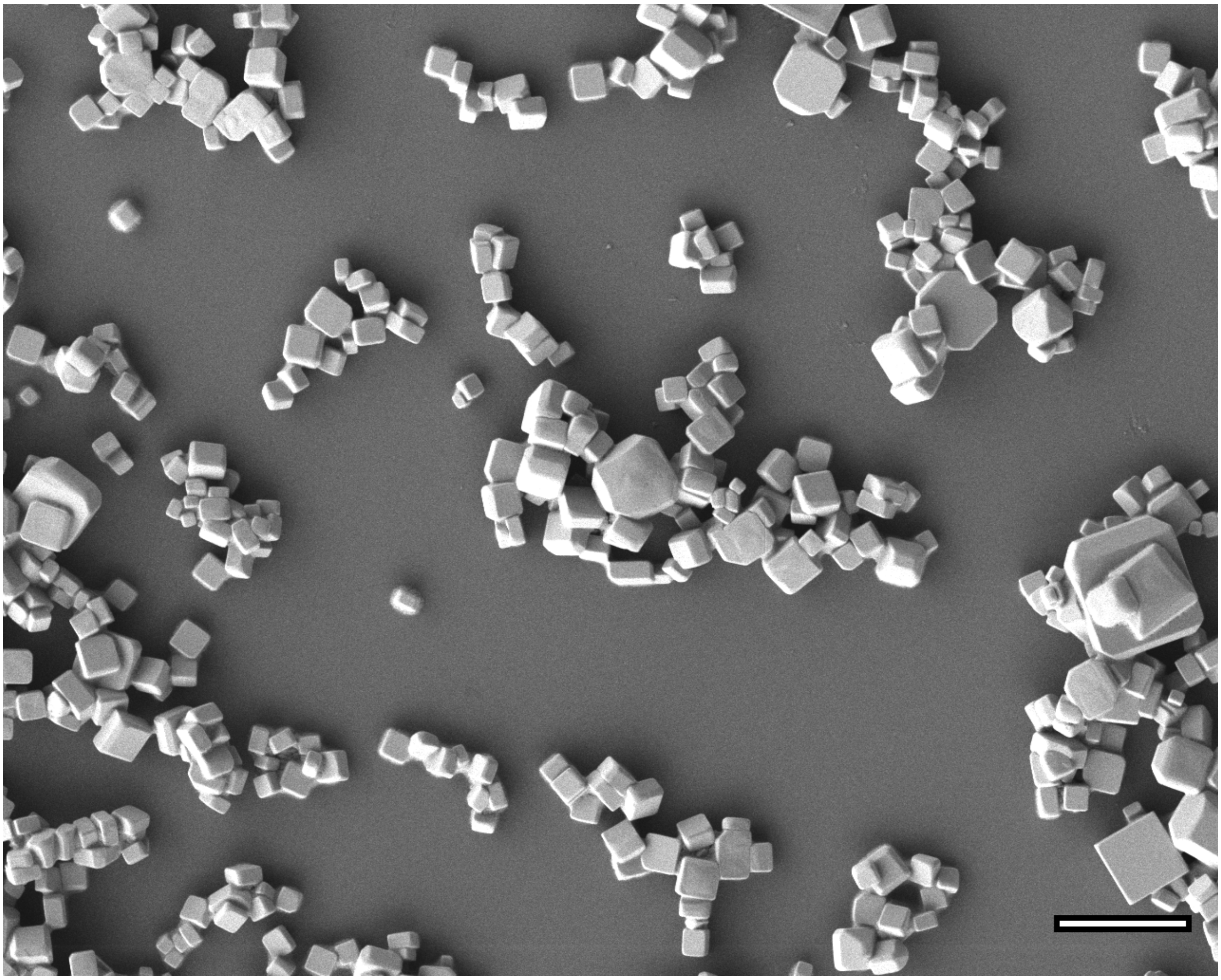

c) cubic particles, scale bar = 5 μm.

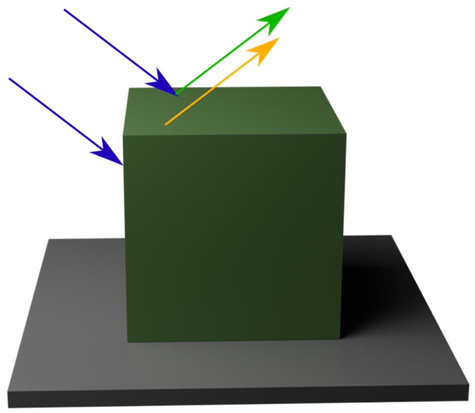

Figure S7. Sketch of self-absorption in 90 ° geometry.

Sketch showing the self-absorption effect for detected light, being traversed through the material yielding in distorted contribution to the spectra.

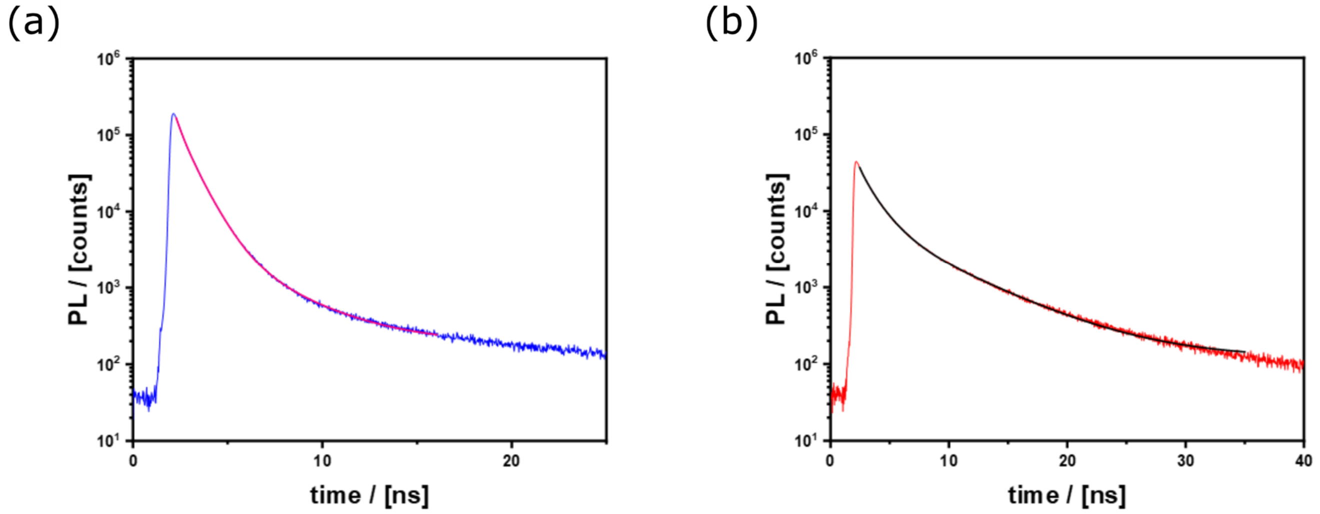

Figure S8. Time resolved PL spectra (ensemble measurements) fitted by a triexponential fit.

PL decay spectra for (a) RD with (110) top facet, and (b) CU, with (100) top facet, particles; both with a triexponential fit, resulting in a lifetime of 3 ns for (110) surface and 6 ns for (100) surface.

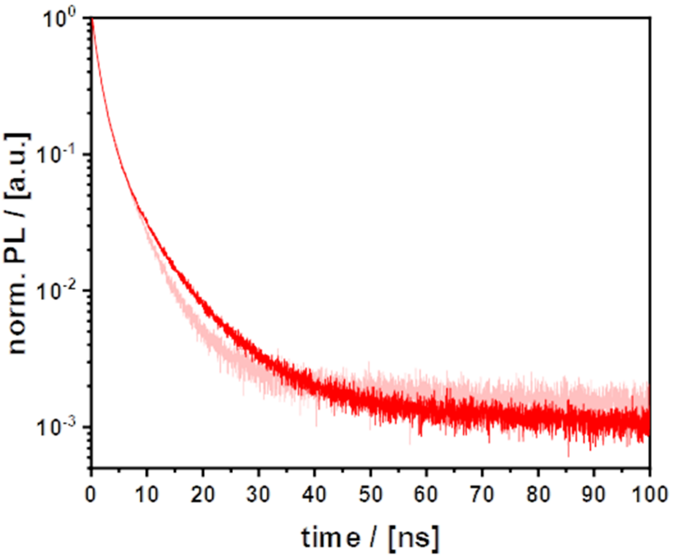

Figure S9. Time resolved PL spectra for CU particles with different sizes.

a) PL decay spectra of cubic shaped particles with different sizes. Deep red curve particles with averaged size of 1775 ± 616 nm, light red curve 5314 ± 1233 nm.

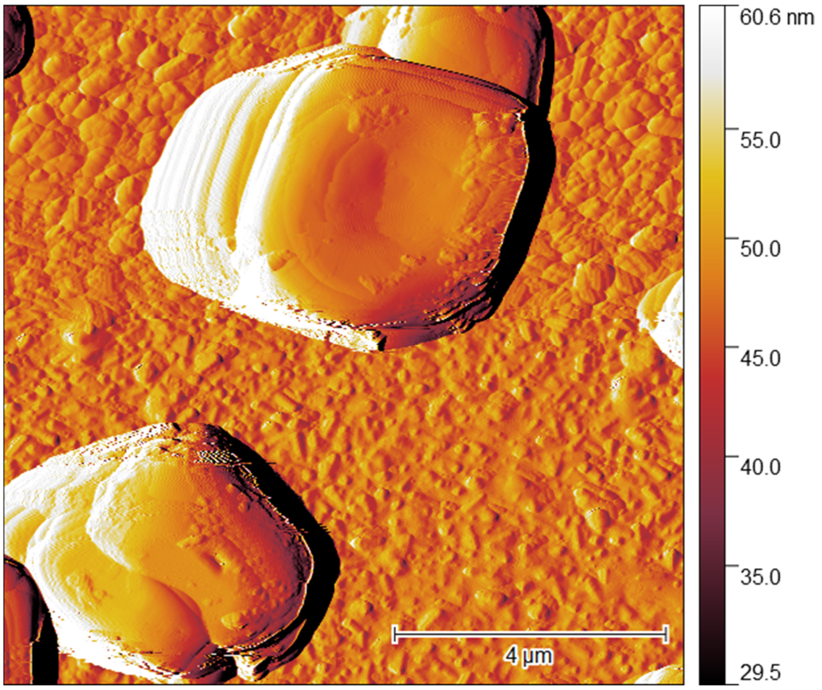

Figure S10. Additional data from KPFM measurements.

a) KPFM (amplitude) images of CU particles, showing the morphology and the top-facet, parallel to the substrate.

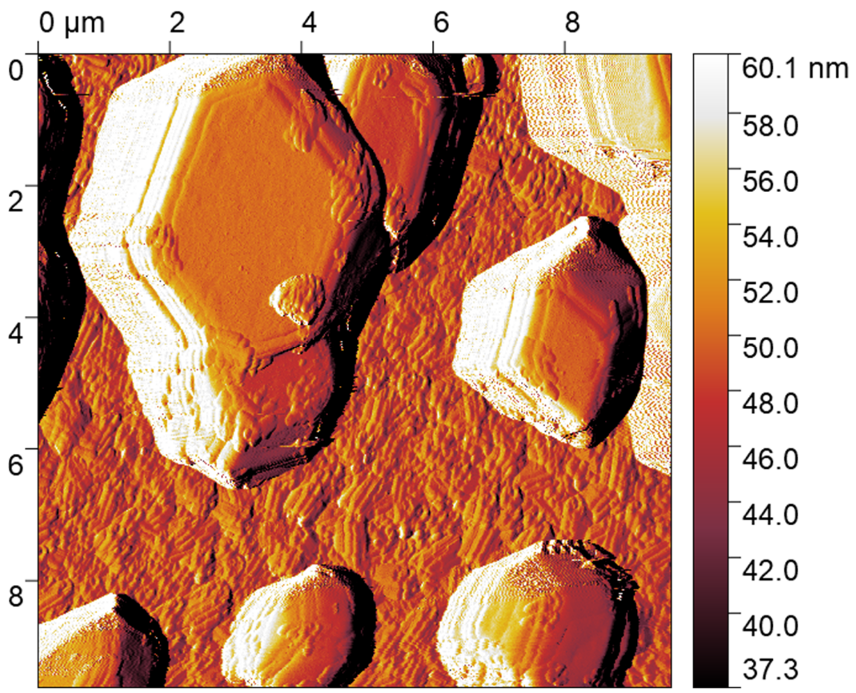

b) KPFM (amplitude) images of RD particles, showing the morphology and the top-facet, parallel to the substrate.

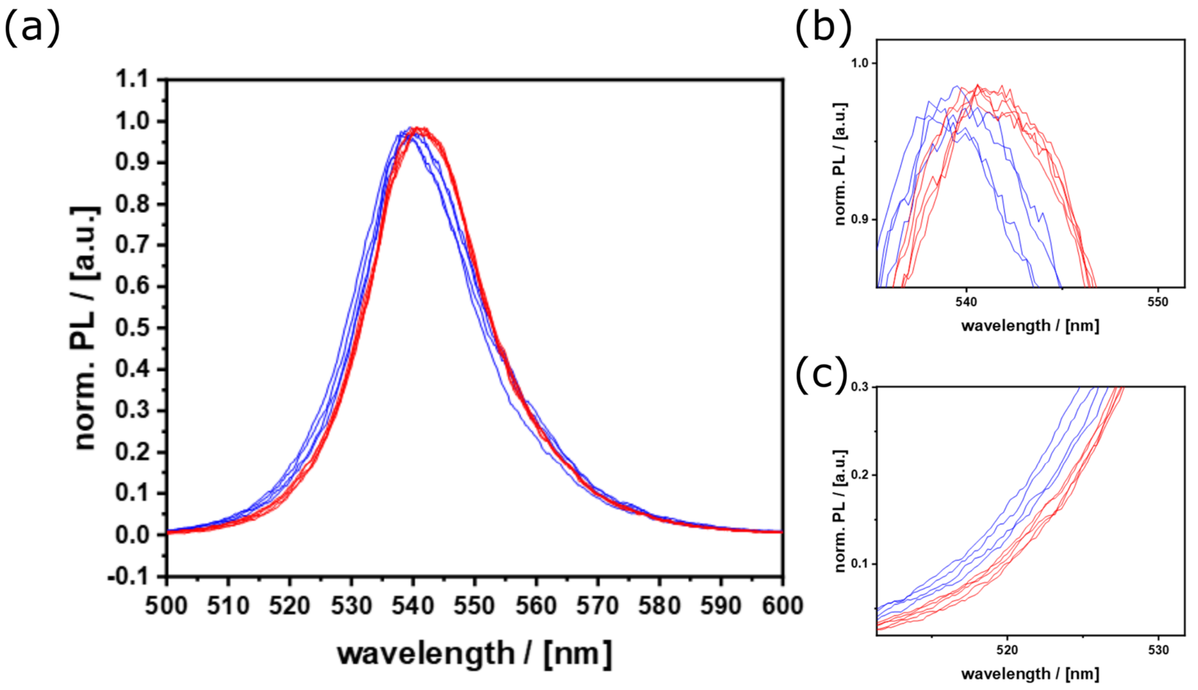

Figure S11. space resolved PL emission spectra for different facets. (**a**) µPL emission spectra measured on different RD (blue) and CU (red) particles with zooms into (**b**) the red shoulder of the peak and (**c**) the high energy side. The measurements confirm the in the main text mentioned effects over every single measurement carried out.
